# Phenazine-Based Homogeneous Photocatalysts for Visible-Light-Driven Hydrogenation of Nitroarenes Under Mild Conditions

**DOI:** 10.3390/molecules31071063

**Published:** 2026-03-24

**Authors:** Van Dao, Thanh Huyen Vuong, Nguyen Kim Nga, Esteban Mejía

**Affiliations:** 1 Leibniz Institute for Catalysis (LIKAT), Albert-Einstein-Str. 29a, 18059 Rostock, Germany; van.dvt511@gmail.com (V.D.); huyen.vuong@catalysis.de (T.H.V.); 2 School of Chemistry and Life Sciences, Hanoi University of Science and Technology, 1 Dai Co Viet Road, Hanoi 100000, Vietnam; nga.nguyenkim@hust.edu.vn

**Keywords:** phenazines, visible-light photocatalysis, nitroarene hydrogenation, metal-free photoredox catalysis, radical reactions

## Abstract

Phenazine derivatives are promising metal-free chromophores with strong redox and photophysical properties, yet their use in photocatalytic hydrogenation remains limited. Here, we report a homogeneous phenazine-based system for the visible-light-driven hydrogenation of nitroarenes under mild conditions. Using nitrobenzene as a model substrate and triethanolamine as a sacrificial hydrogen source, the photocatalyst achieved aniline yields of up to 81% after 12 h of irradiation at 390 nm. Systematic variation in reaction parameters revealed that catalyst structure, solvent, and light wavelength strongly influence performance. Kinetic analysis indicated that prolonged irradiation reduces overall yield due to the reconversion of reactive intermediates. The system exhibited higher efficiency toward nitroarenes bearing electron-withdrawing groups, while aliphatic nitro compounds underwent only partial reduction. Mechanistic studies using UV–Vis, fluorescence, and EPR spectroscopy confirmed the formation of persistent radical species and supported a stepwise electron and proton transfer mechanism. This work showcases the potential of phenazine-based photocatalysts as metal-free platforms for nitroarene reduction under visible light.

## 1. Introduction

The reduction of aromatic nitro compounds to anilines is a fundamental transformation in organic synthesis, owing to the prevalence of arylamine motifs in pharmaceuticals, agrochemicals, and dyes [1,2]. Traditional hydrogenation methods often rely on heterogeneous transition-metal catalysts (e.g., Pd, Pt, Ni) or stoichiometric reductants (e.g., LiAlH_4_, NaBH_4_), which pose limitations in terms of sustainability, selectivity, safety [3,4,5]. In recent years, metal-free catalytic strategies have received attention as more sustainable alternatives. Among these, homogeneous organocatalytic and organic photoredox systems have enabled efficient nitroarene hydrogenations under mild conditions [6,7]. For instance, benzothiazoline-based transfer hydrogenations promoted by Brønsted acids have proven selective and metal-free under thermal conditions [8]. Meanwhile, organic chromophores such as Eosin Y have facilitated visible-light-driven single-electron transfer (SET) pathways, achieving selective nitro group reductions in the presence of sacrificial amines and green-light irradiation. Such systems often show broad substrate scope and high chemoselectivity, driven by well-tuned redox potentials and the photogeneration of nitro radical anions [9].

**Figure 1:**
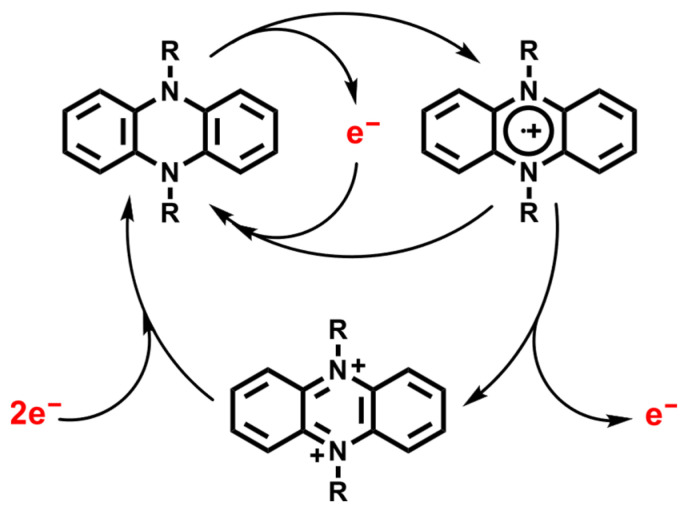
Redox behavior of 5,10-dihydrophenazine derivatives. These molecules readily engage in single-electron transfer (SET) processes to generate highly reactive radical canionic intermediates.

Despite these advances, multi-electron reduction pathways such as nitro-to-aniline conversions remain mechanistically demanding. The process involves sequential proton-coupled electron transfers through nitroso and hydroxylamine intermediates, which are susceptible to side reactions (e.g., azo formation, overreduction) [9]. This presents a challenge for both electron transfer efficiency and intermediate control. Recent progress in organic redox catalyst design offers new tools for tackling such transformations. Notably, phenazine-based catalysts have emerged as a robust platform for redox catalysis due to their reversible electron transfer behavior and structural tunability. Early works in our group demonstrated that N-substituted phenazines act as effective thermal catalysts for aerobic oxidative homo- and cross-coupling of amines [10], and cross-dehydrogenative Aza-Henry reaction [11]. These early contributions established phenazines as redox-flexible, thermally stable organic catalysts for redox processes (Figure 1).

In a pioneering study, Miyake and co-workers introduced 5,10-dihydrophenazine derivatives as highly potent photoredox catalysts capable of promoting photoinduced SET to activate aryl halides and drive bond-forming reactions under visible-light irradiation [12]. Their work underscored the exceptional reducing power of the phenazine excited state, along with its notable photostability and capacity to engage in both oxidative and reductive quenching cycles. Building on this concept, our group recently demonstrated that a range of structurally diverse phenazine derivatives can function as efficient photocatalysts for the reductive dehalogenation of iodoarenes, proceeding through sequential hydrogen atom transfer (HAT) and SET mechanisms [13].

These findings position phenazines as versatile organic photocatalysts that could excel in demanding reductive cycles. Yet their use in nitroarene hydrogenation remains scarce, particularly in homogeneous metal-free contexts. In this study, we explore the potential of phenazine-based photocatalysts for the selective reduction of nitroarenes under visible light and mild conditions, advancing both the mechanistic understanding and synthetic scope of phenazine-enabled photoredox hydrogenations.

## 2. Results and Discussion

**Figure 2:**
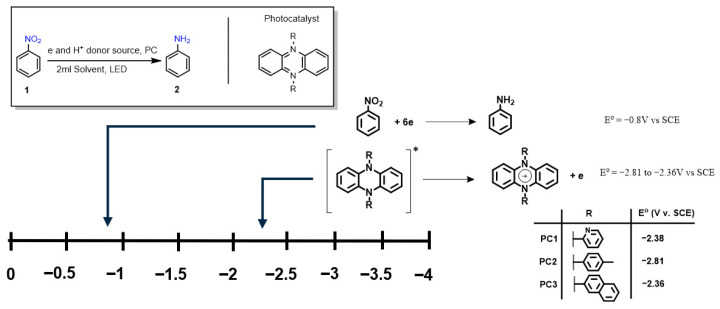
Redox potentials of phenazine-based photocatalysts **PC1**–**PC3** compared to a representative nitroarene substrate (nitrobenzene). The strongly negative potentials support their ability to drive photoinduced electron transfer for nitroarene reduction. The asterisk (*) indicate an electronic excited state.

Phenazine-based photocatalysts previously developed by our group were selected for evaluation as candidates for the sustainable and selective formation of anilines under mild conditions [13]. These catalysts exhibit sufficiently negative redox potentials, enabling the reduction of nitroarene substrates in the presence of a sacrificial electron donor and hydrogen source. To characterize their redox behavior, we employed spectroelectrochemical (SEC) analysis in combination with cyclic voltammetry (CV). The photocatalysts **PC1**, **PC2**, and **PC3** displayed redox potentials of −2.38 V, −2.81 V, and −2.36 V vs. SCE, respectively [13]. These values are markedly more negative than the reported reduction potential of typical nitroarene substrates (−0.8 V vs. SCE) [14], thereby supporting their thermodynamic capacity to initiate substrate reduction via photoinduced electron transfer (Figure 2).

### 2.1. Screening of Reaction Conditions for the Photocatalytic Hydrogenation of Nitrobenzene

The reduction capabilities of **PC1**–**PC3** were evaluated in the photocatalytic hydrogenation of nitrobenzene under argon atmosphere, irradiated with a 390 nm LED at room temperature (Table 1). Among the three photocatalysts tested, **PC1** exhibited slightly superior hydrogenation performance. This enhanced selectivity might be attributed to the electron-withdrawing 2-pyridyl group in **PC1**, which leads to lower-energy excited states with charge-transfer character [13]. Such stabilization facilitates electron transfer and promotes the stepwise cleavage of the N=O π-bonds and formation of N–H bonds. Evidence for reaction intermediates such as azoxy and azo species was supported by GC–MS analysis.

Although **PC3** achieved a high yield (80%), it was excluded from further screening due to its poor atom economy and low synthetic yield. Consequently, **PC1** was identified as the most effective homogeneous catalyst for this transformation and was selected for further optimization. Interestingly, the high yield observed for **PC3** suggests that the naphthyl substituent may exert a weak electron-donating effect, although it does not significantly disrupt the phenazine core’s redox behavior.

Next, the effect of various bases on the photocatalytic hydrogenation of nitrobenzene was investigated, with particular attention to the role of triethanolamine (TEOA) as the sacrificial electron and proton donor (Table 2). While most tested bases supported notable conversion, hydrazine showed no measurable activity, likely due to its high water content, which compromised the solubility of the photocatalyst in what is intended to be a homogeneous reaction mixture. Among the tested amines, TEOA and dimethylaminoethanol (DMAE) led to the highest substrate conversions and aniline yields—81% and 56%, respectively. Also, TEOA outperformed diethanolamine (DEA), which is attributed to its three hydroxyl groups that act as additional proton-donating sites. This structural feature enhances its efficiency as both an electron and proton donor. The electron-donating hydroxyl groups in TEOA not only stabilize cationic intermediates during the catalytic cycle but also facilitate the regeneration of the photocatalyst’s neutral ground state, thereby sustaining the catalytic turnover. Additionally, the release of protons through nucleophilic substitution by hydroxyl-bearing amines occurs more readily than alkylation processes typical of methyl-substituted amines.

Incremental increases in TEOA concentration beyond 3 equivalents did not significantly impact reaction yield. Theoretically, the complete reduction of a nitro group to an aniline requires 6 electrons and 6 protons, which can be supplied by 2 equivalents of TEOA, producing oxidized side-products such as carboxylic acids. The stable yields suggest that this stoichiometry is sufficient under the reaction conditions. Based on these observations, 3 equivalents of TEOA were selected as the optimal condition for all further investigations into nitrobenzene hydrogenation.

A variety of solvents was screened to assess their influence on the photocatalytic reduction of nitrobenzene to aniline (Table 3). Glycerol, a green solvent, was initially considered but showed negligible conversion, likely due to the poor solubility of the photocatalyst in this viscous medium. Another environmentally benign option, formic acid, afforded modest conversion (60%) and yield (44%). However, its acidic nature likely interferes with the basicity of triethanolamine (TEOA), the sacrificial electron donor, potentially inhibiting the reaction or degrading the photocatalyst.

Among the solvents tested, DMSO—known for scavenging hydroxyl radicals (•OH)—delivered high conversion and moderate aniline yield, indicating compatibility with the photocatalytic cycle. However, acetonitrile, a polar aprotic solvent, provided both high conversion and the highest selectivity for aniline formation. In contrast, protic solvents such as isopropanol (i-PrOH) and other alcohols facilitated higher overall conversions. Notably, i-PrOH achieved quantitative conversion and a yield of 81% in the presence of PC1. In this case, i-PrOH may act not only as solvent but also as the hydrogen donor. The reduced hydrogenation yields observed in highly polar protic solvents such as ethanol and formic acid can be primarily attributed to the limited solubility of the phenazine-based photocatalyst under these conditions. This effect was particularly pronounced in the case of glycerol, where complete immiscibility between the catalyst and the solvent resulted in no detectable reaction.

Conversely, despite not contributing reducing equivalents directly, acetonitrile’s high selectivity for aniline made it the solvent of choice in this study, especially given the focus on selective nitroarene hydrogenation.

The effect of irradiation wavelength on photocatalytic activity was evaluated (see Table S1). The photocatalyst demonstrated enhanced nitrobenzene reduction at shorter wavelengths, particularly in the UV range around 370 nm. This correlates with increased light absorption as the excitation wavelength decreased from 420 nm to 320 nm (see Figure S89). Notably, among the tested visible wavelengths, only irradiation at 440 nm resulted in nitrobenzene conversion, alas with only moderate aniline yields, suggesting the involvement of non-productive side reactions. This indicates that the photocatalyst is not effectively excited at wavelengths beyond the 440 nm absorption threshold.

Additional reaction parameters were screened in order to gain a better understanding of the behaviour of the photocatalytic system (see Table S1). In aprotic solvents such as acetonitrile, an inverse correlation was observed between catalyst loading and product yield. Although increasing the photocatalyst concentration to 11 mol% improved conversion, the aniline yield declined sharply to 49%. The decrease in yield is probably due to an increase in the formation of side products, which results from excess catalyst loading causing an imbalance in the reaction steps.

When isopropanol was used as the solvent and hydrogen donor, the system responded differently. Here, conversion remained consistently near 100%, and yield exhibited a nonlinear trend with increasing catalyst loading. The aniline yield remained stable at low catalyst loadings (2–5 mol%), peaked at 93% with 9–11 mol% PC1 and decreased slightly to 80% at 13 mol%. These fluctuations are again attributed to excessive light absorption by surplus photocatalyst hindering the formation of its radical cation, which in turn suppresses the generation of the electrons and protons required for nitroarene reduction.

Control experiments highlighted the essential role of each component: no product formation was observed in the absence of light, or when a suitable hydrogen and electron donor was not present. This confirms that the reaction is not thermally driven. Interestingly, TEOA (a widely used sacrificial electron donor) [15] can be readily oxidised under photoirradiation to form an α-amino radical, which can then abstract hydrogen atoms from isopropanol. This process generates α-hydroxyalkyl radicals, which are subsequently converted to acetone, releasing hydrogen atoms that indirectly facilitate the reduction of the nitro group. This behaviour is consistent with previous reports that highlight the reducing nature of isopropanol, which contributes to increased product yields by acting as both a solvent and a hydrogen source [16]. In contrast, using hydrazine as the electron donor yielded no conversion, mirroring results obtained in the dark. This is likely due to its high water content, which negatively impacts the solubility of the photocatalyst in acetonitrile. Similarly, the presence of oxygen was found to partially inhibit the hydrogenation process, albeit not severely. Finally, the impact of radical scavenging was examined using TEMPO at 5 mol% and 3 equivalents (corresponding to the concentrations of the catalyst and TEOA, respectively). In both cases, photocatalytic activity remained unchanged, suggesting that radical intermediates may have short lifetimes or are not significantly quenched by TEMPO. Fluorescence studies support this observation, indicating that the excited states of the phenazine catalysts and electron donors are transient and decay rapidly under reaction conditions.

### 2.2. Kinetic Profiling and Product Speciation of Nitrobenzene Reduction

**Figure 3:**
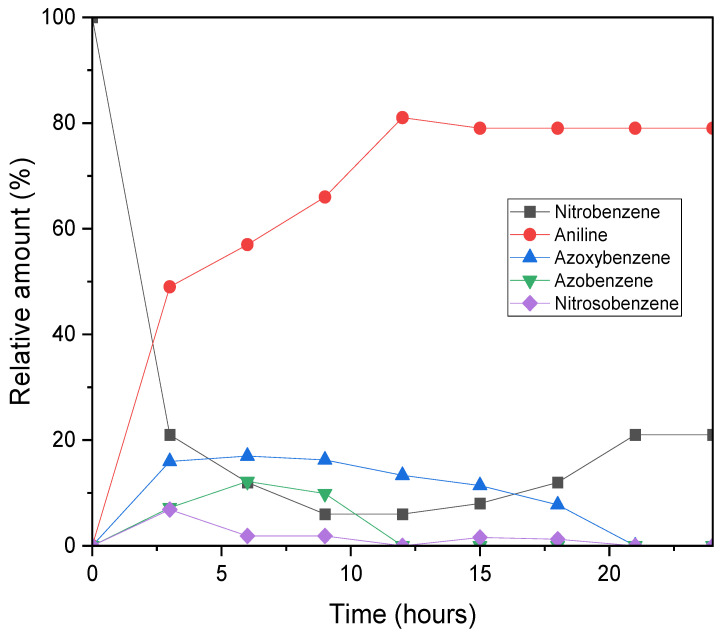
Reaction profile for the photocatalytic reduction of nitrobenzene to aniline using PC1 as the photocatalyst. Reaction conditions: Nitrobenzene (0.5 mmol), PC1 (5 mol%), triethylamine (TEOA) (3 equivalents), acetonitrile (2 mL), under an argon atmosphere, irradiated with a 390 nm LED at 27 °C for 24 h. All conversions and yields were quantified by gas chromatography (GC) analysis using n-octane as the internal standard.

To gain a deeper insight into how reaction intermediates evolve over the 24 h photocatalytic cycle, the reduction profile of nitrobenzene under standard conditions was monitored using GC–MS (see Figure 3). Within the first three hours of irradiation, both conversion and aniline yield increased sharply, reaching 79% conversion and 49% yield, respectively. Aniline formation then continued steadily, peaking at an 81% yield after 12 h, remaining stable thereafter. In contrast, the nitrobenzene conversion profile showed a non-monotonic trend: the total amount of reduced products peaked at 92% after 9 h, but then declined to 79% after 24 h. Nevertheless, the final selectivity towards aniline was complete. This behaviour suggests the transient formation and degradation of unstable intermediates between 0 and 15 h. Some of these intermediates may have undergone reoxidation back to nitrobenzene, potentially via interaction with reactive oxygen species (e.g., superoxide anion radical, O_2_^•−^), which are likely to be generated from water decomposition during the reduction cycle. The stabilisation of the reaction profile after ~22 h likely reflects the depletion of reactive intermediates, which suppresses side reactions and allows the system to reach a steady state [17,18].

Further support for a reversible Haber mechanism [19], where azo intermediates reconvert to nitrobenzene via nitrosobenzene intermediates, was provided by GC–MS analysis. This was evidenced by a drop in azoxybenzene concentration coinciding with an increase in nitrosobenzene, which reinforces the idea of a dynamic equilibrium among the intermediates. Phenylhydroxylamine was notably absent, likely due to its spontaneous condensation with nitrosobenzene and/or dehydration, both of which rapidly convert it into more stable intermediates, such as azoxybenzene. The inherently unstable nature of phenylhydroxylamine, along with its rapid consumption, explains its absence from the GC–MS spectrum (Figure S5). The relatively high abundance of azoxybenzene and nitrosobenzene compared to azobenzene and phenylhydroxylamine can be attributed to their lower activation energy barriers, which allow them to persist for longer in the reaction mixture prior to analysis. These findings are consistent with previous studies, which have identified azoxy- and azobenzene derivatives as key intermediates, or even target products, in nitrobenzene reduction via thermal, chemical, photocatalytic and electrochemical approaches [19,20].

### 2.3. Substrate Scope

**Figure 4:**
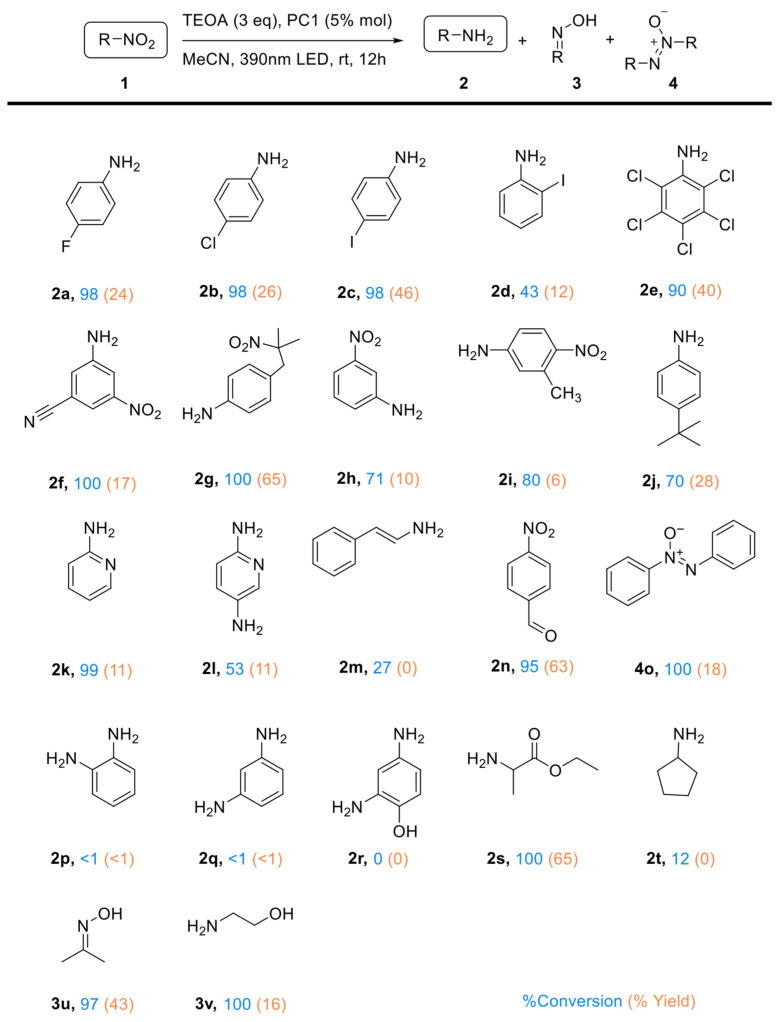
Reaction products from the photocatalytic hydrogenation of nitroarenes and aliphatic nitro compounds using PC1. Reaction conditions: Substrate (0.5 mmol), PC1 (5 mol%), triethylamine (TEOA) (3 equivalents), acetonitrile (2 mL), under an argon atmosphere and irradiated with a 390 nm LED at 27 °C for 12 h. Conversions and yields were determined by gas chromatography–mass spectrometry (GC–MS) analysis using n-octane as the internal standard, based on relative peak areas.

The photocatalytic reductive activity of PC1 was evaluated using various nitroarenes (see Figure 4). Substrates bearing halogen substituents exhibited high reactivity, with conversion rates reaching 98%, alas only with moderate aniline yields (up to 46% for **1c**) Interestingly, aniline yields decreased as halogen electronegativity increased, likely due to stronger electron-withdrawing effects increasing the efficiency of electron transfer from the TEOA^•+^ donor, resulting in the acceleration of unwanted side-reactions. Furthermore, the position of the halogen also had a significant impact: para-substituted 4-iodonitrobenzene (**1c**) demonstrated greater reductive efficiency and generated a wider range of intermediates than its ortho-substituted analogue (**1d**) (Figures S69 and S70). The presence of five chlorine groups in quintozene (**1e**) appears to balance intramolecular electron withdrawal, resulting in higher yields than those of mono-substituted analogues.

Quintozene (**1e**) and 3-iodonitrobenzene (**1c**) were selected for an in-depth analysis of the product profile due to their moderate yields and high conversion rates. In both cases, the reactions proceeded rapidly, with the intermediates being further converted into secondary products. For **1e**, hexachlorobenzene and pentachlorobenzene were detected, with the latter accounting for 45% of the product mixture (see Table S2 and Figures S28–S31 and S71). This suggests a mechanism involving C–N bond cleavage to generate a pentachlorophenyl radical (C_6_Cl_5_^•^), which then either captures a chlorine radical (possibly from residual hydrochloric acid in commercial quintozene) to form hexachlorobenzene, or abstracts a hydrogen atom to yield pentachlorobenzene. In the reduction of **1c**, multiple products were identified, including aniline, nitrobenzene, azobenzene, azoxybenzene and a mono-iodinated azobenzene derivative (see Table S3 and Figures S22–S24).

Beyond halogenated nitroarenes, strong reactivity was also observed for 2-nitro-2-(4-nitrobenzyl)propane (**1g**) and ethyl 2-nitropropionate (**1s**), with full conversion and yields of up to 65% being achieved. Among the dinitrobenzene derivatives tested, the highest conversions were exhibited by 3,5-dinitrobenzonitrile (**1f**), and 2,5-dinitrotoluene (**1i**), while 1,3-dinitrobenzene (**1h**) showed slightly reduced efficiency.

Some substrates did not yield aniline derivatives, instead forming alternative reduced products. For example, trans-β-nitrostyrene (**1m**) exhibited poor reactivity, producing only minor non-Haber-type by-products (Figures S45–S49). Full conversion was achieved in the case of 4-nitrobenzonitrile (**1o**), with the formation of azoxybenzene (18%) and azobenzene (8%) as the major products.

Substrates containing both nitro and amino groups (e.g., **1l**, **1o**, **1p** and **1q**) generally showed negligible conversion. The exception was **1l**, a pyridine derivative, which underwent significant conversion, albeit with a low yield of amine products. Similarly, 2-nitropyridine (**1k**) achieved near-complete conversion, though the yield of the corresponding aniline remained low.

2-nitroethanol (**1v**) and 2-nitropropane (**1u**) underwent high conversion, producing low yields of aminoethanol (16%) for the former, while dimethyl ketoxime was identified as a major product for the later. The bulkier aliphatic substrates, nitrocyclopentane (**1u**), showed only ~12% conversion into minor by-products, with traces of cyclopentanone oxime being detected by GC–MS. (Figures S62 and S86)

Overall, photocatalyst PC1 demonstrated activity towards a broad range of substrates, achieving high conversion efficiencies, and modest selectivities. The structure of the substrate had a significant impact on selectivity, with several compounds undergoing non-Haber-type transformations involving C–X and N–N bond cleavage to form diverse reduction products beyond conventional anilines.

### 2.4. Mechanistic Studies

#### 2.4.1. UV-Vis Absorption Studies

**Figure 5:**
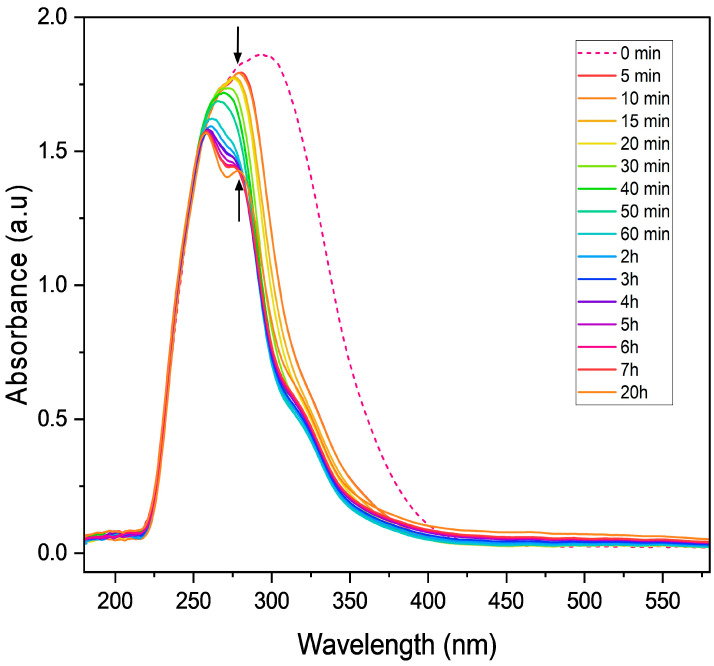
Time-resolved UV–Vis absorption spectra of the photocatalytic mixture containing nitrobenzene (0.08 mmol), PC1 (0.8 mol%), and TEOA (0.5 equiv.) in acetonitrile at 300 K and ambient pressure under continuous 390 nm LED irradiation over 20 h.

Time-resolved UV–Vis spectroscopy was conducted over a 20 h period using a solution containing nitrobenzene (0.08 mmol), TEOA (0.5 equivalents) and PC1 (0.8 mol%) in acetonitrile, under continuous irradiation with a 390 nm LED light source. As illustrated in Figure 5, exposure to violet light caused the broad absorbance feature of the initial mixture to narrow and decrease significantly in intensity within the first 5 min, indicating rapid photoexcitation and the dynamics of the initial reaction stages. Over the next two hours, the absorbance intensity continued to decline and peak splitting was observed, suggesting the emergence of new, distinct species within the system.

These spectral changes likely correspond to the formation of key radical intermediates, including the nitrobenzene radical anion, the PC1 and TEOA radical cations [13], and potentially other transient species involved in the stepwise reduction of the nitro group.

#### 2.4.2. Fluorescence Studies

To further investigate these intermediates, fluorescence spectroscopy was employed to monitor quenching behaviour and emission changes. Consistent with the observations of Enoki, monomeric dye-based photocatalysts tend to exhibit weak fluorescence [21]. Moreover, phenazine derivatives are known to have weak fluorescence due to rapid intersystem crossing (ISC), which leads to a triplet state and subsequent phosphorescence emission. The low singlet–triplet gap in phenazine often results in back triplet–singlet interconversion, which causes thermally activated delayed fluorescence. Thus, emission quenching may be due to several factors, including energy transfer from both excited states, and electron transfer from the triplet state in a catalytic reaction [12,13].

In this study, excess amounts of each component were used deliberately to enhance signal clarity, compensating for the short-lived excited states and rapid radiative decay typical of organic dye photocatalysts compared to metal-based ones [7].

**Figure 6:**
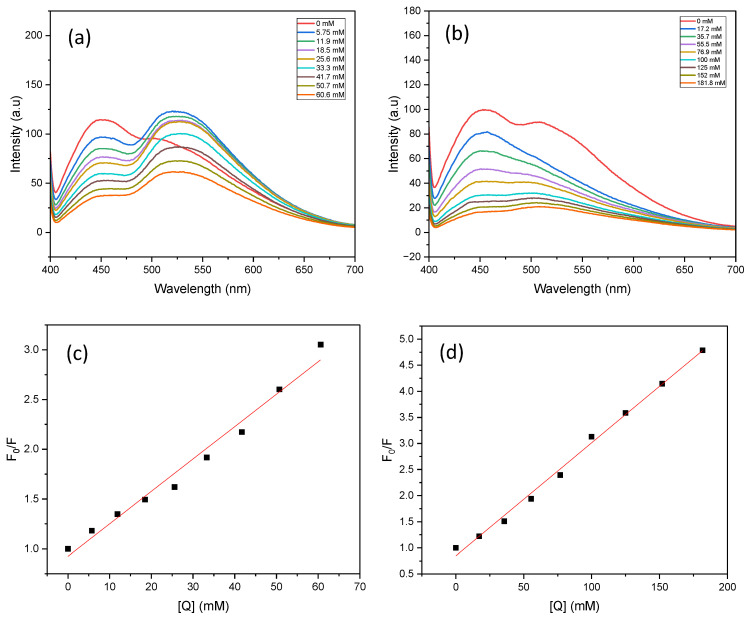
Fluorescence spectra and Stern–Volmer plots of PC1 in the presence of nitrobenzene (NB, (**a**,**c**)) and triethanolamine (TEOA, (**b**,**d**)). Measurements were performed in acetonitrile with 0.03913 mM PC1 under standard conditions, using an excitation wavelength of 390 nm.

The fluorescence spectra of PC1 were recorded to investigate its oxidative and reductive quenching behaviour in the presence of nitrobenzene (NB) and triethanolamine (TEOA), respectively. Excitation wavelengths of 370, 390, 440 and 467 nm were used (See Figure 6 and Figures S90–S92). Fluorescence emission was weakest at 467 nm, with peak intensities below 50 a.u., indicating minimal photocatalyst excitation at this wavelength. As the excitation wavelength decreased, the fluorescence intensity increased and reached a maximum of 124 a.u. at 390 nm. This correlated with the highest aniline yield observed under identical conditions. This suggests that the efficiency of fluorescence quenching is closely linked to photocatalytic activity.

Under 390 nm excitation, PC1 in acetonitrile exhibited an emission peak centred at 466 nm, with a secondary shoulder around 508 nm (Figure 6a). When 5.75 mM nitrobenzene was added, the emission profile changed notably, yielding two distinct peaks: a sharp peak at 448 nm (~98 a.u.) and a broader peak at 523 nm (~124 a.u.). In contrast, the addition of TEOA caused the disappearance of the 508 nm shoulder (Figure 6b), resulting in a simpler spectrum with a single dominant emission. This indicates that there are distinct quenching mechanisms between the two quenchers.

Furthermore, the fluorescence intensity of the PC1 and nitrobenzene mixture was significantly higher than that of the PC1 and TEOA mixture. This suggests that the initial interaction between PC1 and nitrobenzene is stronger. This implies that nitrobenzene is more readily reduced by the excited-state PC1, whereas TEOA primarily acts in the catalyst regeneration step by forming the TEOA radical cation.

The fluorescence quenching data were analysed using the Stern–Volmer model (see Figure 6c,d). For TEOA, the Stern–Volmer plot showed strong linear correlation (R^2^ > 0.99) across all eight tested concentrations, indicating dynamic quenching behaviour. In contrast, the nitrobenzene plot was only linear at lower concentrations, deviating from linearity at higher quencher levels. This non-linearity may be due to saturation effects, inner filter effects, or changes in the local microenvironment surrounding PC1 at elevated nitrobenzene concentrations.

#### 2.4.3. Electronic Paramagnetic Resonance (EPR) Studies

**Figure 7:**
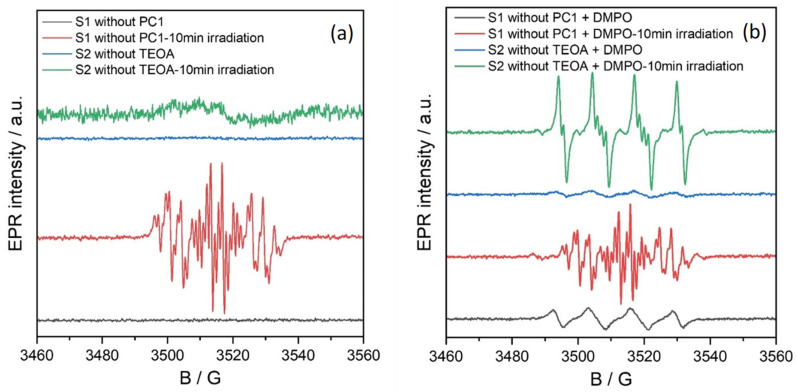
EPR spectra of solutions S1 and S2 (without PC1 and TEOA, respectively) recorded before and after irradiation with a 390 nm LED light source. (**a**) spectra without a spin-trapping agent; (**b**) spectra in the presence of DMPO.

In situ EPR spectroscopy was conducted to identify the key radical species generated during the photocatalytic reduction of nitrobenzene to aniline using PC1. Figure 7 shows the EPR spectra of two control solutions: S1, which lacks PC1 (substrate: 0.5 mmol, TEOA: 3 equivalents, acetonitrile: 2 mL), and S2, which lacks TEOA (substrate: 0.5 mmol, PC1: 5 mol%, acetonitrile: 2 mL). Both samples were measured under an oxygen atmosphere at room temperature in the dark, and after 10 min of irradiation with a 390 nm (40 W) LED lamp.

Under dark conditions, no EPR signals were observed in either solution (black and blue traces in Figure 7), indicating the absence of detectable radical species without photoexcitation. However, upon irradiation, the EPR spectrum of solution S1 (without PC1) displayed a distinct multiline signal centred at g = 2.006, with hyperfine coupling constants A_N_ = 12.57 G, A_H_^o^ = 3.67 G and 3.29 G, A_H_^p^ = 3.60 G, A_H_^m^ = 1.10 G (red trace). This pattern is consistent with the formation of a nitrosobenzene radical anion, in agreement with prior literature reports [22]. In contrast, solution S2 (without TEOA) showed only a weak, broad signal indicative of non-specific organic radical formation.

**Figure 8:**
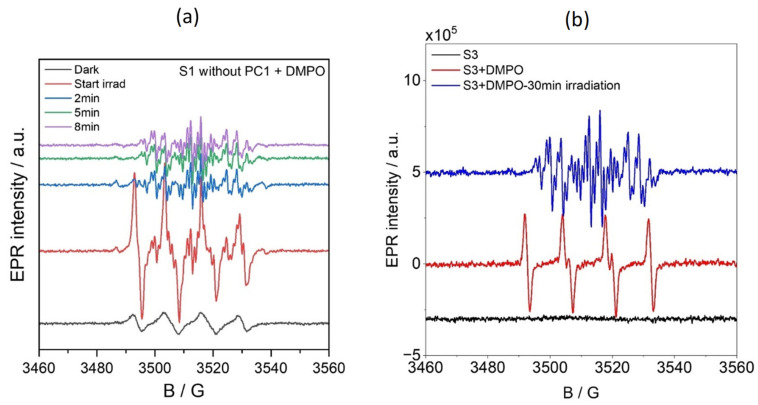
EPR spectra recorded before and during irradiation with a 390 nm LED source of (**a**) solution S1 (standard reaction mixture without PC1) with DMPO, and (**b**) of solution S3 (complete reaction mixture) without and with DMPO.

Spin-trapping experiments were conducted using 5,5-dimethyl-1-pyrroline N-oxide (DMPO) to identify the organic radical species formed during photocatalysis. DMPO was added to solution S2, which contained the substrate and PC1 in acetonitrile but not TEOA. As can be seen in Figure 8 (blue line), the EPR spectrum of this mixture showed a signal that was characteristic of the DMPO/O_2_^•−^ or DMPO/OOH spin adduct, with hyperfine coupling constants of A_N_ = 12.80 G and A_H_^β^ = 10.73 G.

Upon irradiating this solution, a new, intense EPR signal emerged that corresponded to a DMPO/OPh spin adduct, an O-centered organic radical derived from nitrobenzene (not phenoxy) (A_N_ = 12.83 G, A_H_^β^ = 10.17 G and A_H_^γ^ = 1.32 G) [23]. This is shown by the green line in Figure 8 and is confirmed by the simulated spectra in Figure S93b (blue trace). The intensity of this signal decreased with prolonged irradiation (Figure S94), suggesting the transient formation of OPh-centred radicals during the photocatalytic cycle.

A similar DMPO/O_2_^•−^ or DMPO/OOH signal was observed in the S1 solution (substrate and TEOA in acetonitrile, without PC1) under dark conditions (see Figure 8a,b, black lines). Upon in situ irradiation at room temperature, this solution exhibited complex EPR spectra (Figure 8a, red line), indicating the formation of multiple radical species. Simulations of these spectra (Figure S96b) revealed the presence of three distinct spin adducts: a DMPO-C-centred radical, the DMPO-OPh adduct and the nitrosobenzene radical. The corresponding hyperfine parameters are summarised in Table S5. Interestingly, after 10 min of irradiation, only the nitrosobenzene signal remained (Figure 8a, purple line), suggesting that TEOA plays a pivotal role in promoting nitrosobenzene radical formation during photocatalytic reduction.

The reaction mixture S3 (containing all components) exhibited no detectable EPR signal in the dark (Figure 8b, black line). However, when DMPO was added as a spin-trapping agent, a strong signal corresponding to the DMPO/O_2_^−^ or DMPO/OOH adduct appeared (Figure 8b, red line). When this mixture was irradiated in situ at room temperature, the intensity of this signal decreased significantly while new signals corresponding to the DMPO-centred radical adduct and nitrosobenzene radical emerged. After 30 min of continuous irradiation, only the nitrosobenzene signal remained detectable (Figure 8b, blue line), indicating that it is the predominant persistent radical species formed under these reaction conditions.

Taken together, these results confirm the presence of significant radical activity within the system, with the rapid generation of reactive species occurring within seconds of exposure to light. These radicals appear to be highly stable and reactive, making them only partially susceptible to capture by conventional spin-trapping agents.

#### 2.4.4. Proposed Reaction Mechanism

**Figure 9:**
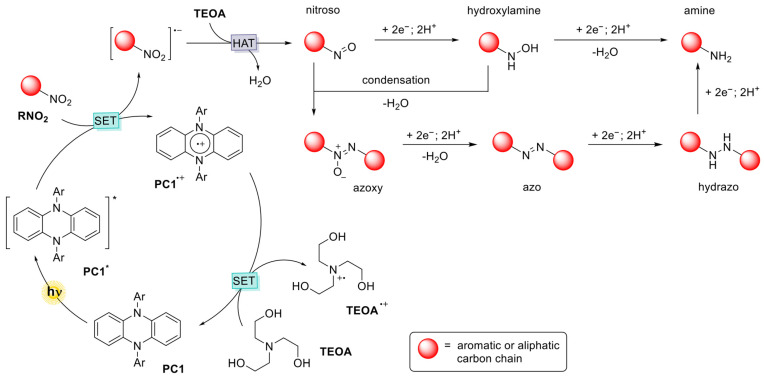
Proposed mechanism for PC1-mediated photocatalytic reduction of nitroarenes under 390 nm light. PC1* undergoes SET with the substrate, forming [RNO_2_]^•−^ and PC1^•+^. TEOA then donates electrons and protons, reducing PC1^•+^ and completing the cycle via Haber’s pathway. The asterisk (*) indicate an electronic excited state.

Based on combined evidence from UV–Vis absorption, fluorescence and EPR spectroscopy, alongside product distribution data and insights from previous studies [10,11,13,20], we propose the following mechanism for the photocatalytic reduction of nitro compounds using phenazine-based catalysts (Figure 9). When irradiated at 390 nm, the photocatalyst PC1 transitions from its ground state to the short-lived singlet excited state. This then rapidly transforms into the long-lived triplet state (PC1*) via intersystem crossing, which enables it to initiate electron transfer processes. The excited PC1* then undergoes photoinduced electron transfer with the substrate (RNO_2_), resulting in the oxidative quenching of the photocatalyst and reduction of the substrate. This step generates the radical cation PC1^•+^ and the radical anion [RNO_2_]^•−^.

The electron-rich [RNO_2_]^•−^ intermediate then proceeds along Haber’s classical hydrogenation pathway, undergoing sequential proton and electron transfers to ultimately form aniline. These protons and electrons are supplied by triethanolamine (TEOA), which simultaneously serves as a sacrificial electron donor. The oxidised photocatalyst PC1^•+^ is subsequently reduced back to its ground state by TEOA, thus closing the catalytic cycle. During this process, TEOA is oxidised to form a radical cation and one of its hydroxyl groups is further oxidised to form a carboxylic acid, releasing two protons that facilitate hydrogenation. GC–MS analysis confirmed the formation of TEOA oxidation by-products, further substantiating its role as an active participant in the reduction mechanism.

## 3. Materials and Methods

All reactions were carried out under an argon atmosphere using standard Schlenk-line techniques, ensuring no exposure to ambient air. The photocatalysts used in this study were synthesized according to a previously reported procedure [13]. A comprehensive description of experimental procedures, materials, and analytical methods is provided in the Supporting Information.

### General Procedure for the Photocatalyzed Reduction of Nitrocompounds

Photocatalytic reactions were conducted in 10 mL glass test tubes containing 2 mL of solvent, into which the photocatalyst, base and substrate (nitrobenzene) were dissolved. The quantities of each component were adjusted according to the specific experimental conditions and recorded precisely to enable the subsequent determination of substrate conversion and product yield. Each test tube was sealed with a rubber septum and purged with argon for 30 min prior to irradiation.

Irradiation was performed using a 52 W Kessil PR160L LED (Richmond, CA, USA) light source with a wavelength of 390 nm and 75% intensity (purple). After the reaction, the homogeneous mixture was left to stand for five minutes and then shaken to ensure uniformity. A 60 µL aliquot was withdrawn using a micropipette, diluted with 1.25 mL of methanol and 17 µL of n-octane (used as an internal standard) and analysed by GC for quantitative determination.

Kinetic studies were carried out under identical conditions, with samples collected at defined time intervals. The same sampling and analytical procedure was employed for substrate scope investigations. Conversion and product yields were determined directly from GC–MS analysis.

## 4. Conclusions

In this study, we developed and evaluated a homogeneous phenazine-based photocatalyst system for the visible-light-driven reduction of nitroarenes and aliphatic nitro compounds under mild conditions. Using triethanolamine as a sacrificial hydrogen and electron donor in acetonitrile, the photocatalytic hydrogenation of nitrobenzene yielded up to 81% aniline after 12 h of irradiation.

Systematic screening of reaction parameters, including substrate structure, solvent, electron donor, photocatalyst loading, and light wavelength, demonstrated their strong influence on reaction efficiency. Kinetic analysis indicated that extended irradiation times can diminish product yields due to the degradation or reconversion of reactive intermediates, such as azo compounds.

Substrate scope experiments revealed that nitroarenes bearing electron-withdrawing groups were efficiently reduced, whereas most aliphatic nitro compounds gave low yields or only partially reduced intermediates (e.g., =N–OH species). Spectroscopic studies (UV–Vis, fluorescence, and EPR) confirmed the light-induced formation of radical intermediates such as, which persisted even in the presence of spin traps.

Taken together, these results establish a promising proof-of-concept for the use of phenazine-based dyes as metal-free photocatalysts for nitroarene reduction. While the observed activity is encouraging, the selectivity toward exclusive aniline formation remains limited, and side-product formation persists in many cases. In perspective, improving both selectivity and substrate scope will require further optimization of the photocatalyst structure and a deeper understanding of reaction conditions and intermediate speciation.

## Figures and Tables

**Table 1:** Photocatalytic hydrogenation of nitrobenzene using phenazine-based photocatalysts ^1^.

Entry	Photocatalyst	Conversion of Nitrobenzene (mol%)	Yield of Aniline (mol%)
1	PC1	>99	81
2	PC2	>99	68
3	PC3	>99	80

^1^ Reaction conditions: Nitrobenzene (0.5 mmol), PC (5 mol%), TEOA (3eq), Solvent: i-PrOH (2 mL) under Argon atmosphere, irradiated by LED 390 nm at temperature of 27 °C for 24 h. All conversions and yields were determined by GC.

**Table 2:** Base screening for the photocatalytic hydrogenation of nitrobenzene ^1^.

Entry	Base	Amount (eq)	Conversion of Nitrobenzene (mol%)	Yield of Aniline (mol%)
1	TEA	3	98	47
2	DIPEA	3	96	55
3	TEOA	3	>99	81
4	TAP	3	51	51
5	DMAE	3	>99	56
6	DEA	3	63	53
7 ^2^	N_2_H_4_•H_2_O	3/6/9	0	0
8	TEOA	5	>99	80
9	TEOA	7	>99	81
10	TEOA	9	>99	82

^1^ Reaction conditions: Nitrobenzene (0.5 mmol), PC1 (5 mol%), Solvent: i-PrOH (2 mL) under Argon atmosphere, irradiated by LED 390 nm at 27 °C for 24 h. All conversions and Yields were determined by GC. ^2^ Solvent: Acetonitrile.

**Table 3:** Solvent screening for the photocatalytic hydrogenation of nitrobenzene ^1^.

Entry	Solvent	Conversion of Nitrobenzene (mol%)	Yield of Aniline (mol%)
1	Methanol	90	55
2	Acetonitrile	79	79
3	DMSO	91	51
4	Iso-propanol	>99	81
5	Glycerol	trace	trace
6	Ethanol	82	66
7	Formic Acid	60	44

^1^ Reaction conditions: Nitrobenzene (0.5 mmol), PC1 (5 mol%), TEOA (3eq), Solvent (2 mL) under Argon atmosphere, irradiated by LED 390 nm at temperature of 27 °C for 24 h. All conversions and yields were determined by GC.

## Data Availability

All raw research data is available from the authors upon request.

## Supplementary Materials

The following supporting information can be downloaded at: https://www.mdpi.com/article/10.3390/molecules31071063/s1, Synthesis of photocatalysts; photocatalytic experiments; gas chromatography; gas chromatography–mass spectrometry; UV-Vis spectroscopy; fluorescence spectroscopy; EPR spectroscopy; NMR spectra of the photocatalysts. Several references are included [13,24,25,26,27,28,29,30,31,32,33].

## Abbreviations

The following abbreviations are used in this manuscript:

CV	cyclic voltammetry
DEA	diethylamine
DIPEA	diisopropylethylamine
DMAE	dimethylaminoethanol
DMPO	5,5-dimethyl-1-pyrroline N-oxide
DMSO	dimethyl sulfoxide
EPR	electron paramagnetic resonance
GCMS	gas chromatography–mass spectrometry
HAT	hydrogen atom transfer
LED	light emitting diode
NB	nitrobenzene
PC	phenazine-based photocatalysts
SEC	spectroelectrochemical analysis
SET	single-electron transfer
SOMO	singly occupied molecular orbital
TAP	triaminopyrimidine
TEA	triethylamine
TEOA	triethanolamine
UV-Vis	ultraviolet-visible spectroscopy
iPrOH	isopropanol
