# Supplementary material for: Phenazine-Based Homogeneous Photocatalysts for Visible-Light-Driven Hydrogenation of Nitroarenes Under Mild Conditions
Source: Molecules. 2026 Mar 24;31(7):1063. doi: 10.3390/molecules31071063 (PMC13074353; doi:10.3390/molecules31071063)
Supplement: Supplementary file 1 [file molecules-31-01063-s001.zip › molecules-4169042-supplementary.pdf]

SUPPORTING INFORMATION

Phenazine-Based Homogeneous Photocatalysts for Visible-Light-Driven Hydrogenation of Nitroarenes under Mild Conditions

Van Dao, Thanh Huyen Vuong, Nguyen Kim Nga and Esteban Mejía

Contents

1. Synthesis of Photocatalysts........2

2. Photocatalytic Experiments........2

3. Gas Chromatography........4

4. Gas Chromatography-Mass Spectroscopy........5

5. UV-Vis Spectroscopy........36

6. Fluorescence Spectroscopy........37

7. EPR Spectroscopy........39

8. NMR Spectra of the Photocatalysts........42

References........46

1. Synthesis of Photocatalysts

All reactions were performed under Argon atmosphere using standard Schlenk-line Technique without any contact to external atmosphere. The synthesis of catalysts in this study was carried out following the reported method.[1]

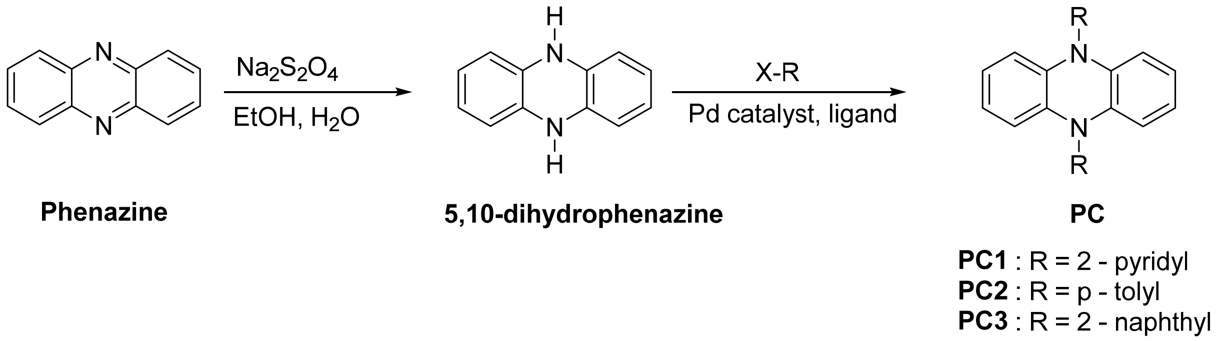

Figure S1. The scheme for the synthesis of the studied photocatalysts.

2. Photocatalytic Experiments

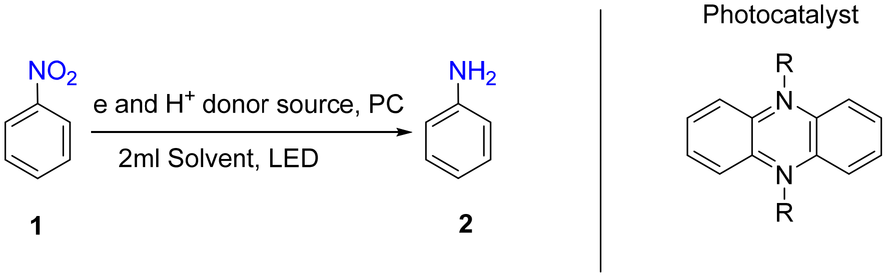

Figure S2. Scheme for the typical photocatalytic reaction

Photocatalytic reactions were conducted in 10 mL glass test tubes containing 2 mL of solvent, in which the photocatalyst, base, and substrate were dissolved. The quantities of each component were adjusted according to the specific experimental conditions and recorded precisely for subsequent determination of substrate conversion and product yield. Each test tube was sealed with a rubber septum and purged with argon for 30 minutes prior to irradiation.

Irradiation was performed using a Kessil PR160L LED light source (52 W, λ = 390 nm, 75% intensity, purple). Following the reaction, the homogeneous mixture was allowed to stand for 5 minutes and then shaken to ensure uniformity. A 60 µL aliquot was withdrawn using a

micropipette, diluted with 1.25 mL of methanol and 17 µL of n-octane (used as an internal standard), and analyzed by GC for quantitative determination.

Kinetic studies were carried out under identical conditions, with samples collected at defined time intervals. For substrate scope investigations, the same sampling and analytical procedure was employed. Conversion and product yields were determined directly from GC–MS analysis.

Table S1. Conditions' screening experiments

Entry	Condition	Conversion[a] (mol%)	Yield[a] (mol%)
Adjusting the amount of PC1			
1	5 mol% [b]	>99	81
2	7 mol% [b]	>99	81
3	9 mol% [b]	98	93
4	11 mol% [b]	98	93
5	13 mol% [b]	96	80
6	5 mol%	79	79
7	7 mol%	>99	55
8	9 mol%	>99	54
9	11 mol%	>99	49
Deviation from the standard conditions			
10	No Cat [b]	>99	70
11	No Cat [e]	>99	77
12	$N_2H_4.H_2O$ replace TEOA	0	0
13	No Light	0	0
14	With Radical Scavenger [b,c]	97	79
15	With Radical Scavenger [b,d]	95	79
16	Under Oxygen Condition	99	60
17	LED 370 nm	86	79
18	LED 390 nm	79	79

19	LED 440 nm	92	45
20	LED 467 nm	0	0

Standard reaction conditions: Nitrobenzene (0.5 mmol), PC1 (5 mol%), TEOA (3eq), Acetonitrile (2ml) under Argon Condition, irradiated by LED 390nm, in the course of 24 hours, at temperature of 27°C. [a] All conversions and Yields were determined by GC. [b] 15 hours [c] 5 mol% of TEMPO was used. [d] 3 equivalents of TEMPO was used . [e] i-Propanol was used as solvent.

3. Gas Chromatography

To determine the conversion and yield rate in each nitrobenzene hydrogenation experiment, a quantitative GC method with an internal standard was employed as the main analytical technique. GC analysis was performed on a Hewlett-Packard 6890 Series instrument, with *n*-octane used as the internal standard. Calibration curves were constructed by plotting the peak area ratio (analyte/*n*-octane) against the corresponding molar ratio of analyte to *n*-octane. This approach enabled quantitative determination of the reaction products with high accuracy and reproducibility. The linearity of the calibration curves was verified with correlation coefficients of R^2 > 0.999, ensuring reliable calculation of conversion and yield values. Calibration curves were generated for both the substrate (nitrobenzene) and the product (aniline) using commercially purchased nitrobenzene and distilled, commercially purchased aniline as analytical reference standards, with *n*-octane serving as the internal standard.

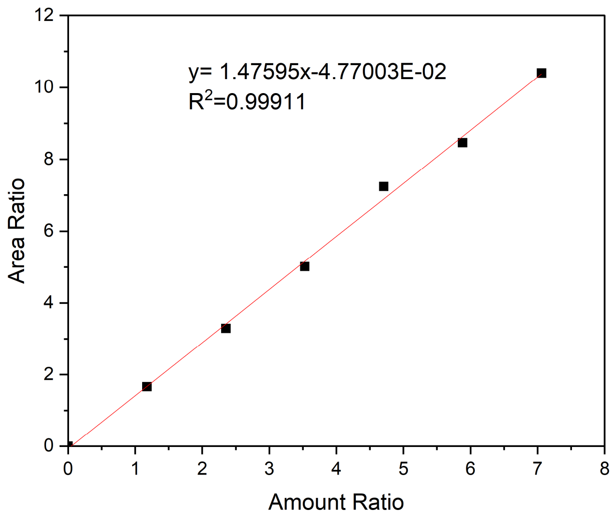

Figure S3. Calibration curve of $PhNH_2$ using n-octane as internal standard

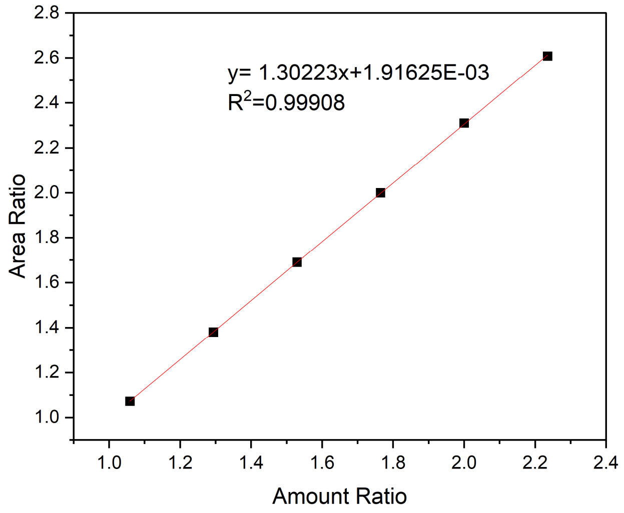

Figure S4. Calibration curve of $PhNO_2$ using n-octane as internal standard

4. Gas Chromatography-Mass Spectroscopy

The components of the product mixtures were determined by GC–MS using *n*-octane as the internal standard. Prior to analysis, it was confirmed that the signal/peak area corresponding to 1 mmol of *n*-octane was comparable to those of the nitroarene compounds (see Supporting Information, Figure IV.1). On this basis, GC–MS was employed to determine both the yield and conversion of the reactions. Conversions and product yields were calculated directly from the GC–MS data by comparing the peak areas of the analytes with that of the known amount of internal standard within the same chromatogram.

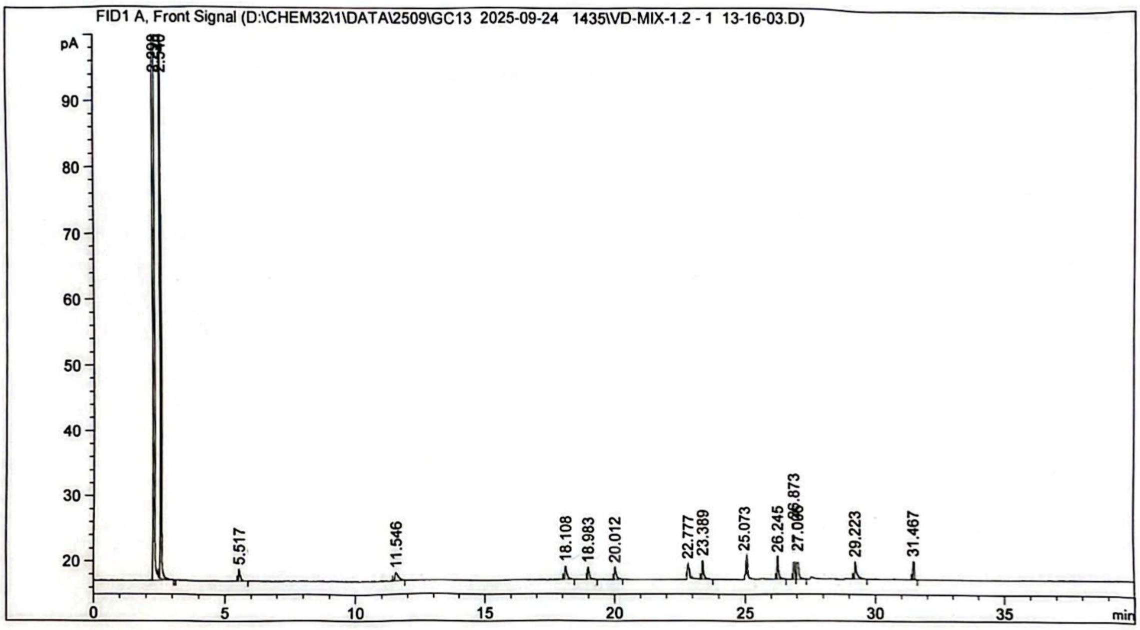

Peak #	RetTime [min]	Type	Width [min]	Area [pA*s]	Height [pA]	Area %	
1	2.298	BB	0.0429	2.66E+04	1.11E+04	93.6185	Methanol
2	2.54	VB	0.0186	1655.27698	1420.62488	5.83457	Acetonitrile
3	5.517	BB	0.0625	11.25338	2.2345	0.03967	1v
4	11.546	BB	0.1284	11.98809	1.25539	0.04226	n-octane
5	18.108	BB	0.0763	11.41185	2.07053	0.04022	1u
6	18.983	BB	0.0659	11.96459	2.32865	0.04317	1t
7	20.012	BB	0.0605	12.01598	2.77682	0.04335	1a
8	22.777	BB	0.0827	11.78369	1.23257	0.04153	1k
9	23.289	BB	0.0554	11.51734	2.96082	0.04060	1b
10	25.073	BB	0.0651	11.98051	1.26702	0.04323	1n
11	26.245	BB	0.0376	11.83657	4.83053	0.04272	1p
12	26.873	BV	0.0392	11.31229	4.51569	0.04087	1j
13	27.006	VB	0.0597	11.20184	7.35678	0.03948	1c
14	29.223	BB	0.0741	11.94487	2.24506	0.04210	1f
15	31.467	BB	0.0409	12.09546	4.71179	0.04263	1e

Figure S5. GC-MS spectra of mixture containing 1mmol of n-octane and several Nitrosoarenes.

For the hydrogenation of compounds 1c, 1d, and 1e, the product mixtures were examined in detail, and the identified components together with their relative amounts are summarized in the accompanying tables. The remaining product mixtures were analyzed under the same protocol to identify additional constituents. Compound identities were mostly assigned by comparing the mass spectra with entries available in several databases and with published literature sources. In cases where no suitable reference could be located, the corresponding

component is noted as not previously documented in the literature. Representative GC–MS spectra of the identified products from the mixtures are provided below.

Table S2. Quintozene **(1e)** reduction reaction's product mixture

**Intermediates**	**Amount (%)**
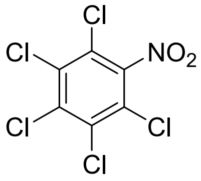	10%
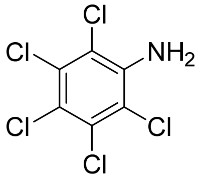	38%
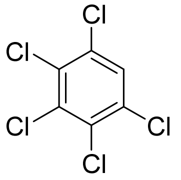	45%
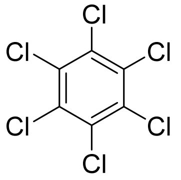	7%

Table S3. 4-iodonitrobenzene **(1c)** reduction reaction's product mixture

**Intermediates**	**Amount (%)**
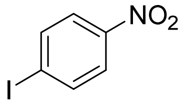	6%
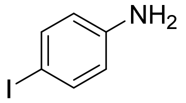	66%
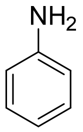	15%
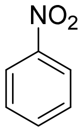	6%
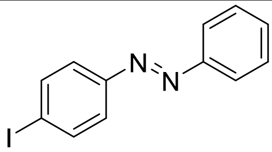	5%
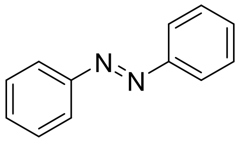	5%
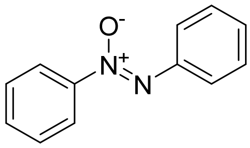	1%

Table S4. 1-iodo-2-nitrobenzene **(1d)** reduction reaction's product mixture

**Intermediates**	**Amount (%)**
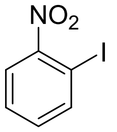	57%
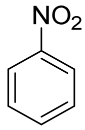	19%
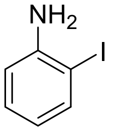	12%
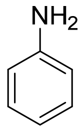	8%
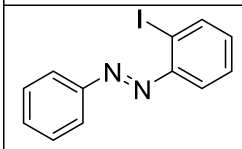	3%
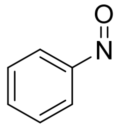	1%

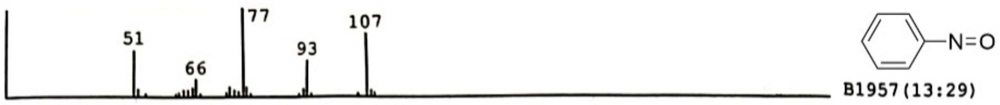

Figure S6. GC-MS spectra of ***Nitrosobenzene*** in the product mixture from the Hydrogenation of Nitrobenzene.

The Mass spectra of this compound has been previously reported in NIST Mass Spectrometry Data Center.[2]

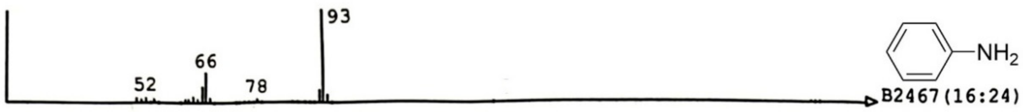

Figure S7. GC-MS spectra of ***Aniline*** in the product mixture from the Hydrogenation of Nitrobenzene.

The Mass spectra of this compound has been previously reported in NIST Mass Spectrometry Data Center. [2]

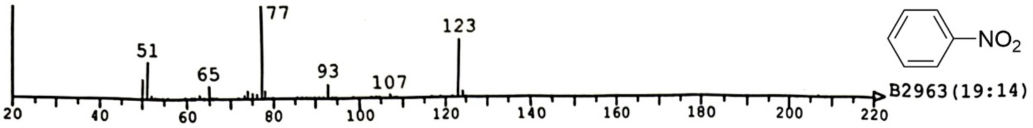

Figure S8. GC-MS spectra of ***Nitrobenzene*** in the product mixture from the Hydrogenation of Nitrobenzene.

The Mass spectra of this compound has been previously reported in NIST Mass Spectrometry Data Center. [2]

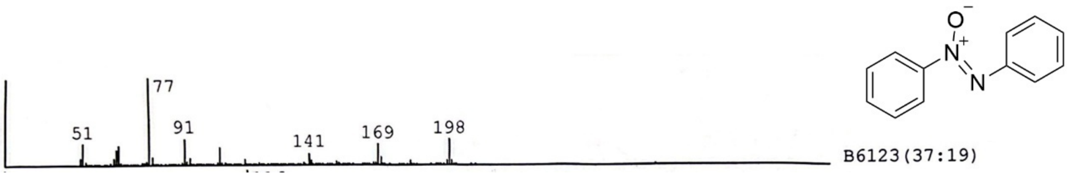

Figure S9. GC-MS spectra of ***Azoxybenzene*** in the product mixture from the Hydrogenation of Nitrobenzene.

The Mass spectra of this compound has been previously reported in NIST Mass Spectrometry Data Center. [2]

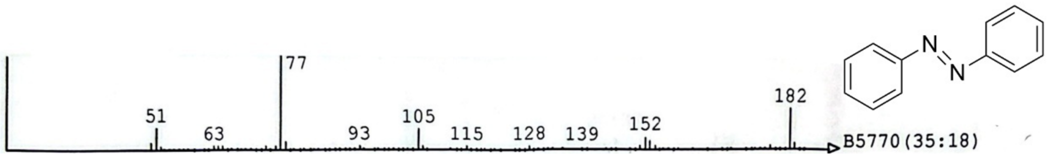

Figure S10. GC-MS spectra of ***Azobenzene*** in the product mixture from the Hydrogenation of Nitrobenzene.

The Mass spectra of this compound has been previously reported in NIST Mass Spectrometry Data Center. [2]

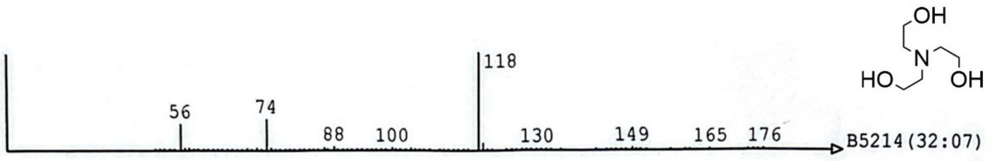

Figure S11. GC-MS spectra of Triethanolamine in the product mixture from the Hydrogenation of Nitrobenzene.

The Mass spectra of this compound has been previously reported in NIST Mass Spectrometry Data Center.[2]

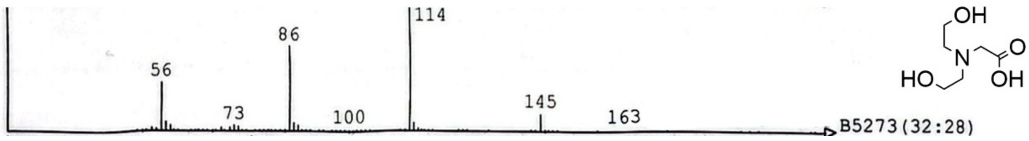

Figure S12. GC-MS spectra of Bicine in the product mixture from the Hydrogenation of Nitrobenzene.

The Mass spectra of this compound has been previously reported on ChemicalBook (https://www.chemicalbook.com/SpectrumEN_150-25-4_MS.htm).

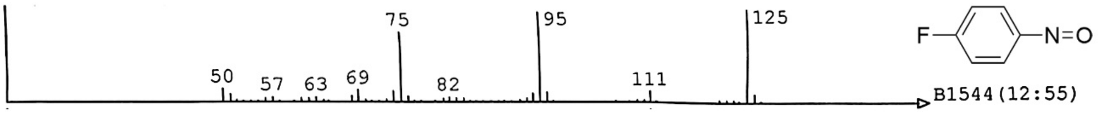

Figure S13. GC-MS spectra of ***1-fluoro-4-nitrosobenzene*** in the product mixture from the Hydrogenation of 1-Fluoro-4-nitrobenzene **(1a)**.

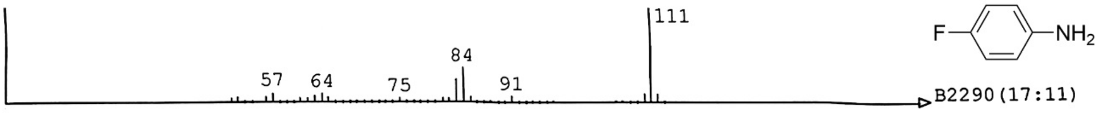

Figure S14. GC-MS spectra of ***4-Fluoroaniline*** in the product mixture from the Hydrogenation of 1-Fluoro-4-nitrobenzene **(1a)**.

The Mass spectra of this compound has been previously reported in NIST Mass Spectrometry Data Center. [2]

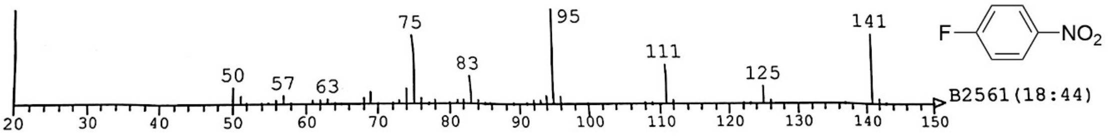

Figure S15. GC-MS spectra of ***1-Fluoro-4-nitrobenzene*** in the product mixture from the Hydrogenation of 1-Fluoro-4-nitrobenzene **(1a)**.

The Mass spectra of this compound has been previously reported in NIST Mass Spectrometry Data Center. [2]

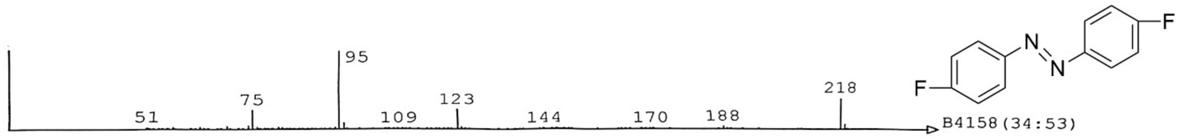

Figure S16. GC-MS spectra of ***4,4'-Difluoroazobenzene*** in the product mixture from the Hydrogenation of 1-Fluoro-4-nitrobenzene **(1a)**.

The Mass spectra of this compound has been previously reported.[3]

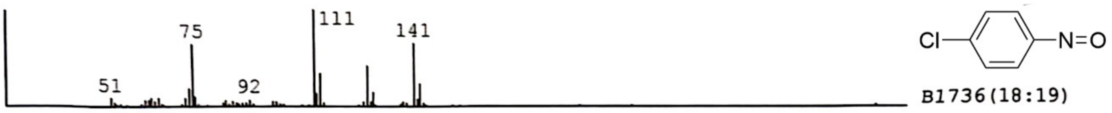

Figure S17. GC-MS spectra of ***1-Chloro-4-nitrosobenzene*** in the product mixture from the Hydrogenation of 1-Chloro-4-nitrobenzene **(1b)**.

The Mass spectra of this compound has been previously reported.[4]

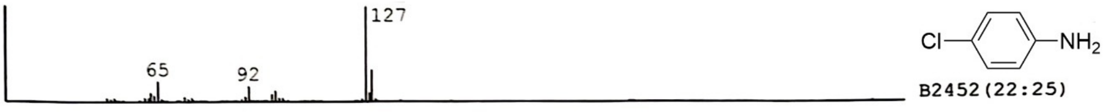

Figure S18. GC-MS spectra of ***4-Chloroaniline*** in the product mixture from the Hydrogenation of 1-Chloro-4-nitrobenzene **(1b)**.

The Mass spectra of this compound has been previously reported in NIST Mass Spectrometry Data Center. [2]

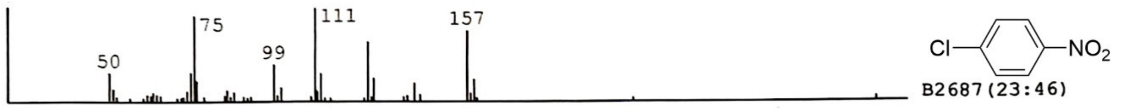

Figure S19. GC-MS spectra of ***1-Chloro-4-nitrobenzene*** in the product mixture from the Hydrogenation of 1-Chloro-4-nitrobenzene **(1b)**.

The Mass spectra of this compound has been previously reported in NIST Mass Spectrometry Data Center. [2]

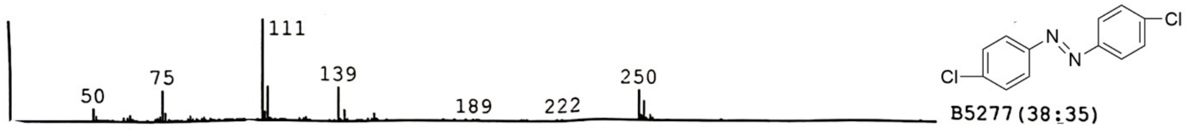

Figure S20. GC-MS spectra of ***4,4'-Dichloroazobenzene*** in the product mixture from the Hydrogenation of 1-Chloro-4-nitrobenzene **(1b)**.

The Mass spectra of this compound has been previously reported in NIST Mass Spectrometry Data Center. [2]

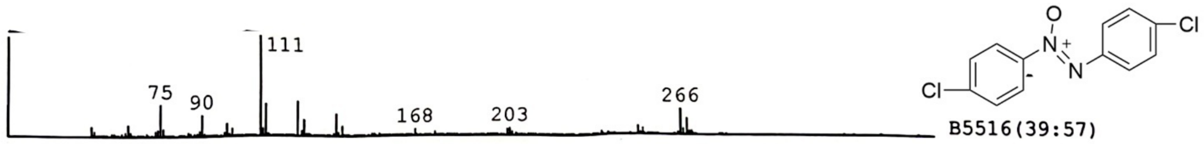

Figure S21. GC-MS spectra of ***4,4'-Dichloroazoxybenzene*** in the product mixture from the Hydrogenation of 1-Chloro-4-nitrobenzene **(1b)**.

The Mass spectra of this compound has been previously reported.[5]

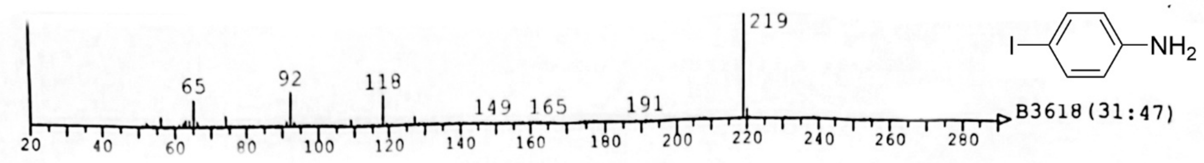

Figure S22. GC-MS spectra of ***4-Iodoaniline*** in the product mixture from the Hydrogenation of 1-Iodo-4-nitrobenzene **(1c)**.

The Mass spectra of this compound has been previously reported in NIST Mass Spectrometry Data Center. [2]

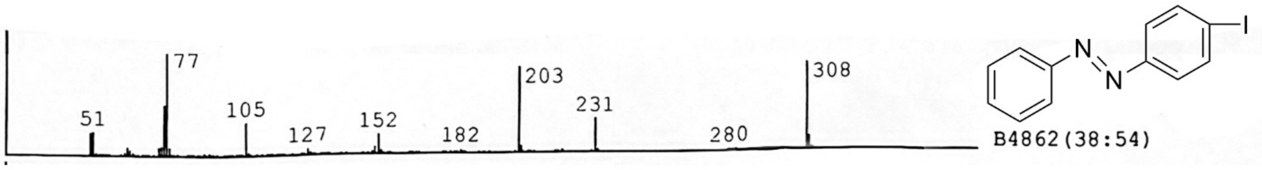

Figure S23. GC-MS spectra of ***(E)-1-(4-iodophenyl)-2-phenyldiazene*** in the product mixture from the Hydrogenation of 1-Iodo-4-nitrobenzene **(1c)**.

The Mass spectra of this compound has been previously reported.[6]

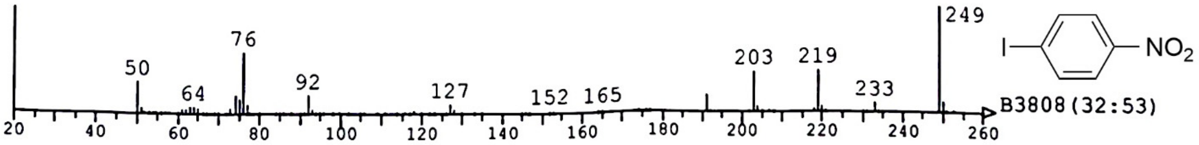

Figure S24. GC-MS spectra of ***4-Iodonitrobenzene*** in the product mixture from the Hydrogenation of 1-Iodo-4-nitrobenzene **(1c)**.

The Mass spectra of this compound has been previously reported in NIST Mass Spectrometry Data Center. [2]

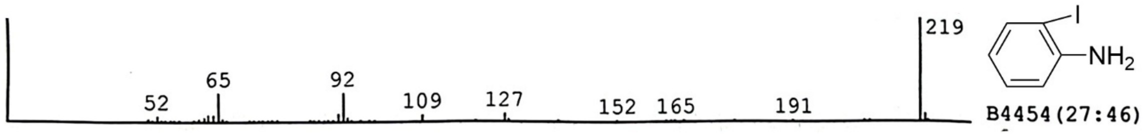

Figure S25. GC-MS spectra of ***2-Iodoaniline*** in the product mixture from the Hydrogenation of 1-Iodo-2-nitrobenzene **(1d)**.

The Mass spectra of this compound has been previously reported in NIST Mass Spectrometry Data Center. [2]

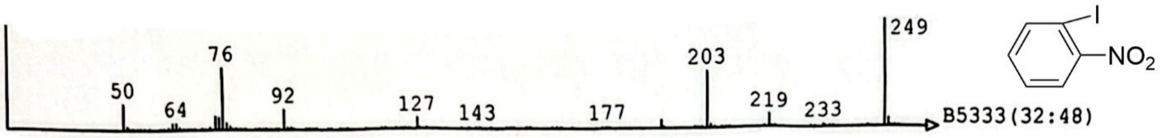

Figure S26. GC-MS spectra of ***2-Iodonitrobenzene*** in the product mixture from the Hydrogenation of 1-Iodo-2-nitrobenzene **(1d)**.

The Mass spectra of this compound has been previously reported in NIST Mass Spectrometry Data Center. [2]

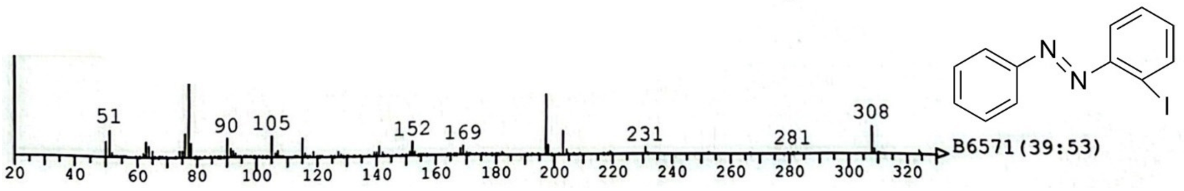

Figure S27. GC-MS spectra of ***(E)-1-(2-iodophenyl)-2-phenyldiazene*** in the product mixture from the Hydrogenation of 1-Iodo-2-nitrobenzene **(1d)**.

The Mass spectra of this compound has been previously reported.[7]

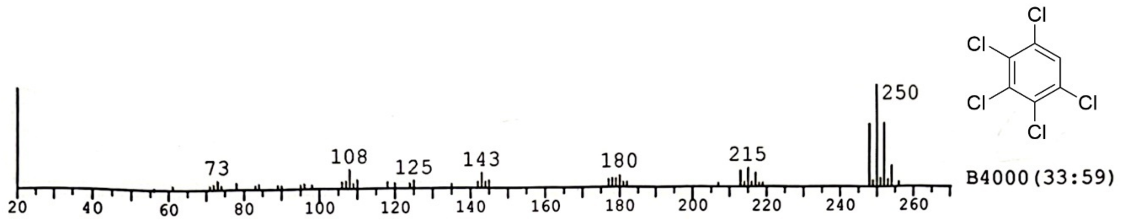

Figure S28. GC-MS spectra of ***Pentachlorobenzene*** in the product mixture from the ssHydrogenation of Quinozene/Pentachloronitrobenzene **(1e)**.

The Mass spectra of this compound has been previously reported in NIST Mass Spectrometry Data Center. [2]

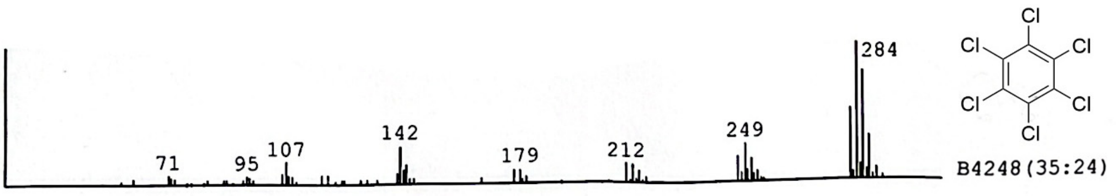

Figure S29. GC-MS spectra of ***Hexachlorobenzene*** in the product mixture from the Hydrogenation of Quinozene/Pentachloronitrobenzene **(1e)**.

The Mass spectra of this compound has been previously reported in NIST Mass Spectrometry Data Center. [2]

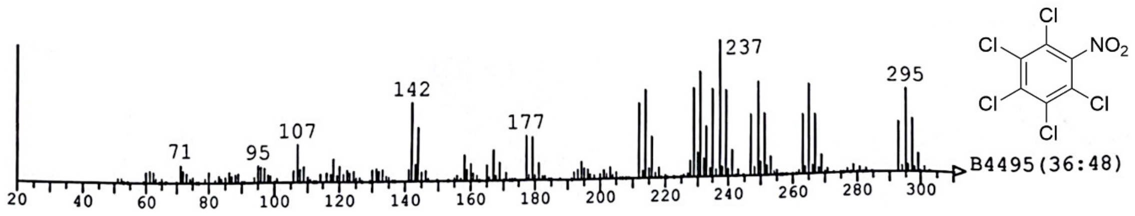

Figure S30. GC-MS spectra of ***Pentachloronitrobenzene*** in the product mixture from the Hydrogenation of Quinozene/Pentachloronitrobenzene **(1e)**.

The Mass spectra of this compound has been previously reported in NIST Mass Spectrometry Data Center. [2]

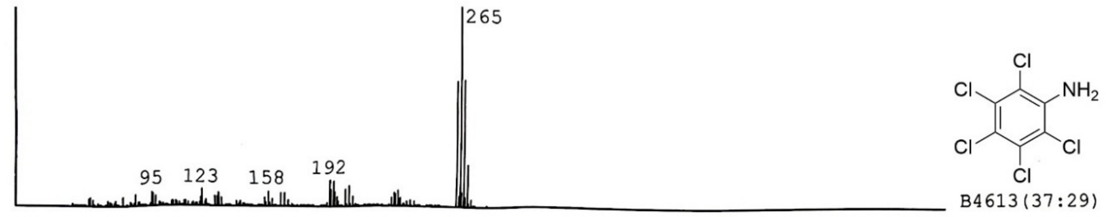

Figure S31. GC-MS spectra of ***Pentachloroaniline*** in the product mixture from the Hydrogenation of Quinozene/Pentachloronitrobenzene **(1e)**.

The Mass spectra of this compound has been previously reported in NIST Mass Spectrometry Data Center. [2]

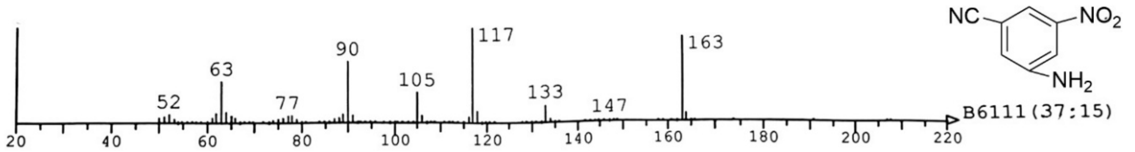

Figure S32. GC-MS spectra of ***3-Amino-5-nitrobenzonitrile*** in the product mixture from the Hydrogenation of 3,5-Dinitrobenzonitrile **(1f)**.

The Mass spectra of this compound has been previously reported in NIST Mass Spectrometry Data Center. [2]

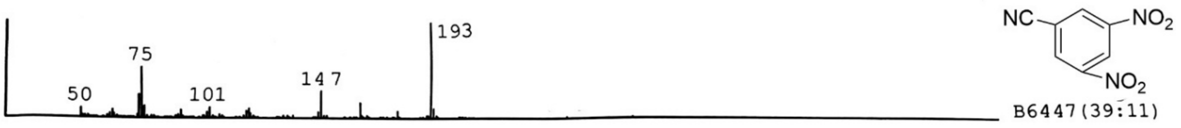

Figure S33. GC-MS spectra of ***3,5-Dinitrobenzonitrile*** in the product mixture from the Hydrogenation of 3,5-Dinitrobenzonitrile **(1f)**.

The Mass spectra of this compound has been previously reported in NIST Mass Spectrometry Data Center. [2]

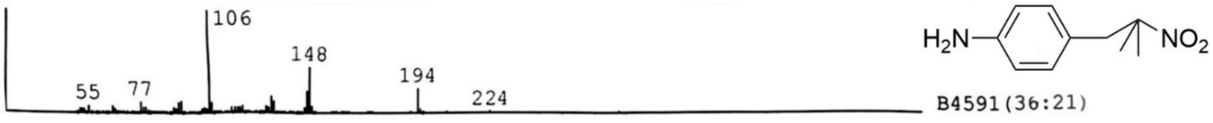

Figure S34. GC-MS spectra of **2-Nitro-2-(4-aminobenzyl)propane** in the product mixture from the Hydrogenation of 2-Nitro-2-(4-nitrobenzyl)propane **(1g)**.

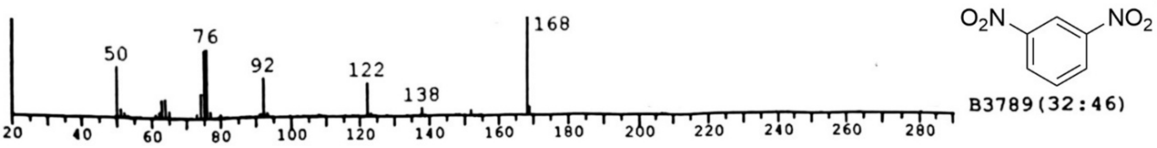

Figure S35. GC-MS spectra of ***1,3-Dinitrobenzene*** in the product mixture from the Hydrogenation of 1,3-Dinitrobenzene **(1h)**.

The Mass spectra of this compound has been previously reported in NIST Mass Spectrometry Data Center. [2]

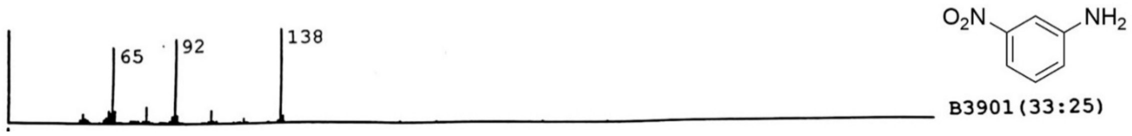

Figure S36. GC-MS spectra of ***3-Nitroaniline*** in the product mixture from the Hydrogenation of 1,3-Dinitrobenzene **(1h)**.

The Mass spectra of this compound has been previously reported in NIST Mass Spectrometry Data Center. [2]

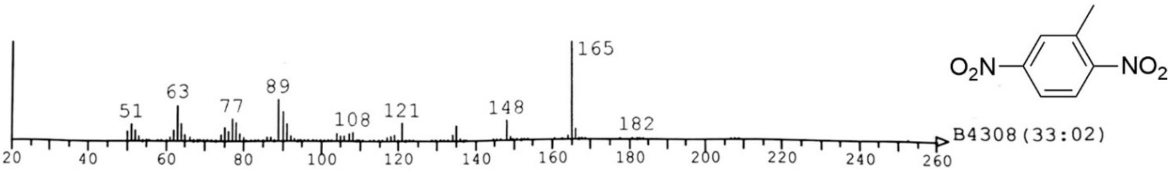

Figure S37. GC-MS spectra of ***2,5-Dinitrotoluene*** in the product mixture from the Hydrogenation of 2,5-Dinitrotoluene **(1i)**.

The Mass spectra of this compound has been previously reported in NIST Mass Spectrometry Data Center. [2]

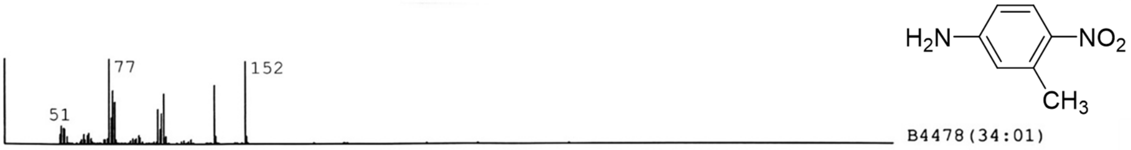

Figure S38. GC-MS spectra of ***3-Methyl-4-nitroaniline*** in the product mixture from the Hydrogenation of 2,5-Dinitrotoluene **(1i)**.

The Mass spectra of this compound has been previously reported.[8]

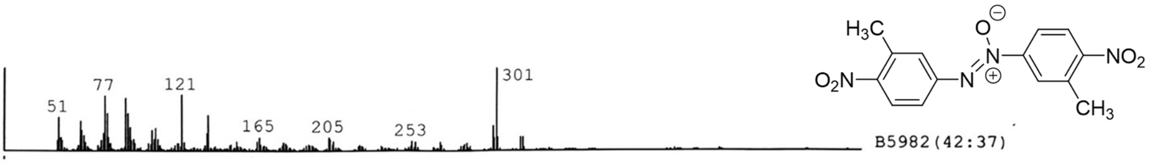

Figure S39. GC-MS spectra of ***(Z)-1,2-bis(3-methyl-4-nitrophenyl)diazene 1-oxide*** in the product mixture from the Hydrogenation of 2,5-Dinitrotoluene **(1i)**.

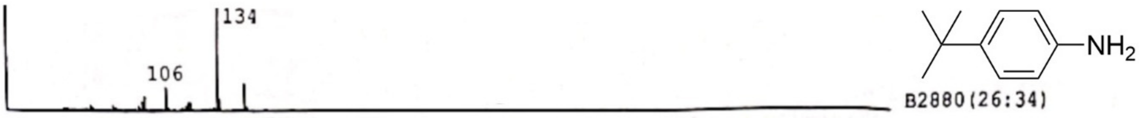

Figure S40. GC-MS spectra of ***1-tert-Butyl-4-aniline*** in the product mixture from the Hydrogenation of 1-tert-Butyl-4-nitrobenzene **(1j)**.

The Mass spectra of this compound has been previously reported in NIST Mass Spectrometry Data Center. [2]

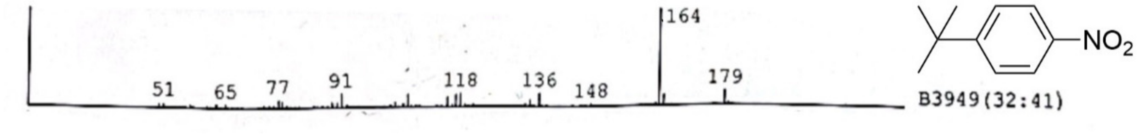

Figure S41. GC-MS spectra of ***1-tert-Butyl-4-nitrobenzene*** in the product mixture from the Hydrogenation of 1-tert-Butyl-4-nitrobenzene **(1j)**.

The Mass spectra of this compound has been previously reported in NIST Mass Spectrometry Data Center. [2]

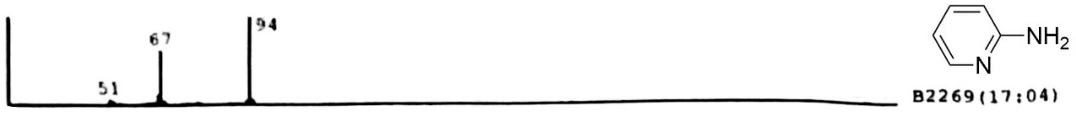

Figure S42. GC-MS spectra of ***2-Aminopyridine*** in the product mixture from the Hydrogenation of 2-Nitropyridine **(1k)**.

The Mass spectra of this compound has been previously reported in NIST Mass Spectrometry Data Center. [2]

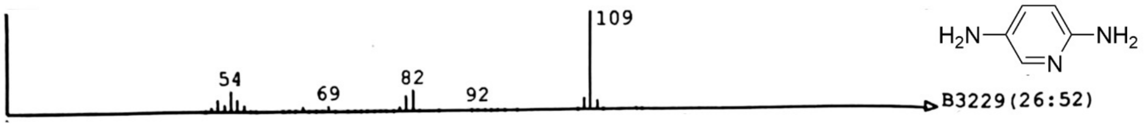

Figure S43. GC-MS spectra of ***2,5-diaminopyridine*** in the product mixture from the Hydrogenation of 2-Amino-5-Nitropyridine **(1l)**.

The Mass spectra of this compound has been previously reported.[9]

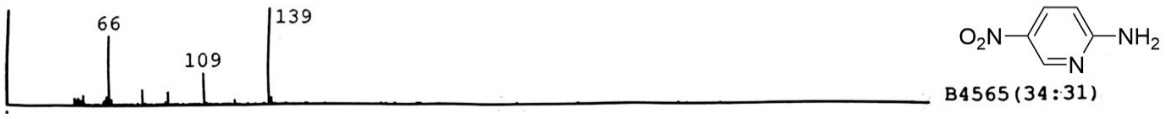

Figure S44. GC-MS spectra of ***2-Amino-5-nitropyridine*** in the product mixture from the Hydrogenation of 2-Amino-5-Nitropyridine **(1l)**.

The Mass spectra of this compound has been previously reported in NIST Mass Spectrometry Data Center. [2]

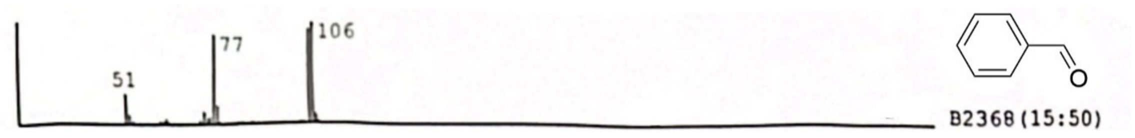

Figure S45. GC-MS spectra of ***Benzaldehyde*** in the product mixture from the Hydrogenation of Trans-β-nitrostyrene **(1m)**.

The Mass spectra of this compound has been previously reported in NIST Mass Spectrometry Data Center. [2]

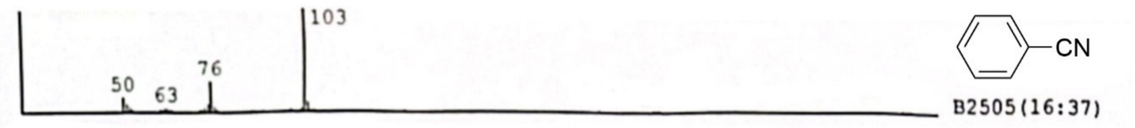

Figure S46. GC-MS spectra of ***Benzonitrile*** in the product mixture from the Hydrogenation of Trans-β-nitrostyrene **(1m)**.

The Mass spectra of this compound has been previously reported in NIST Mass Spectrometry Data Center. [2]

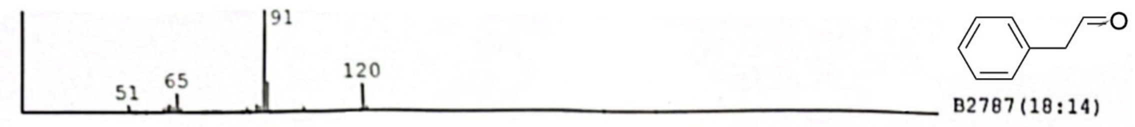

Figure S47. GC-MS spectra of ***Phenylacetaldehyde*** in the product mixture from the Hydrogenation of Trans-β-nitrostyrene **(1m)**.

The Mass spectra of this compound has been previously reported in NIST Mass Spectrometry Data Center. [2]

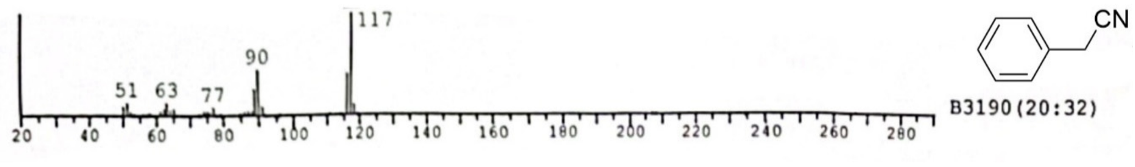

Figure S48. GC-MS spectra of ***Phenylacetonitrile*** in the product mixture from the Hydrogenation of Trans-β-nitrostyrene **(1m)**.

The Mass spectra of this compound has been previously reported in NIST Mass Spectrometry Data Center. [2]

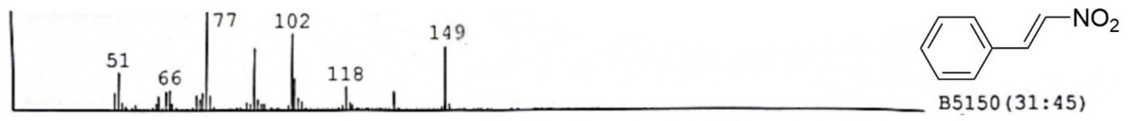

Figure S49. GC-MS spectra of ***Trans- β -nitrostyrene*** in the product mixture from the Hydrogenation of Trans-β-nitrostyrene **(1m)**.

The Mass spectra of this compound has been previously reported in NIST Mass Spectrometry Data Center. [2]

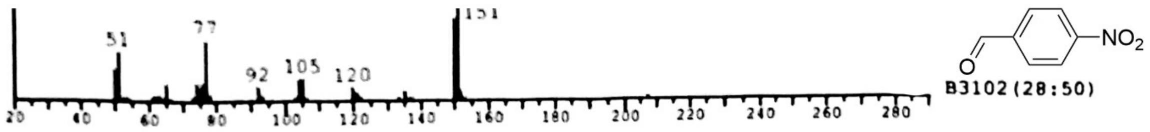

Figure S50. GC-MS spectra of ***4-Nitrobenzaldehyde*** in the product mixture from the Hydrogenation of 4-Nitrobenzaldehyde **(1n)**.

The Mass spectra of this compound has been previously reported in NIST Mass Spectrometry Data Center. [2]

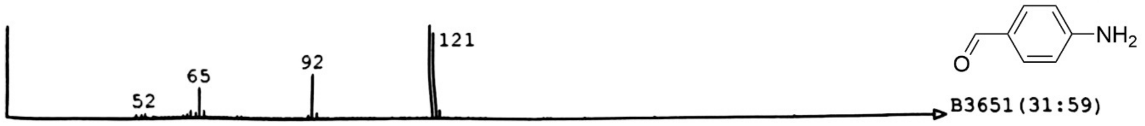

Figure S51. GC-MS spectra of ***4-Aminobenzaldehyde*** in the product mixture from the Hydrogenation of 4-Nitrobenzaldehyde **(1n)**.

The Mass spectra of this compound has been previously reported.[10]

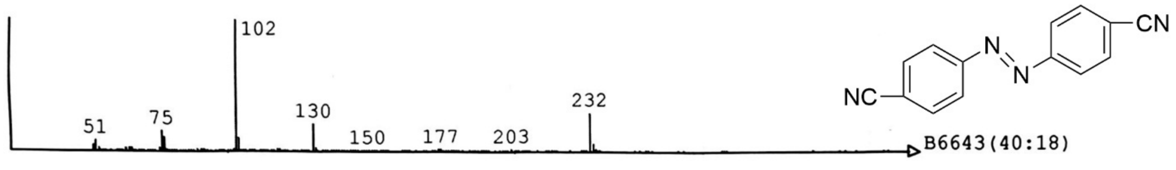

Figure S52. GC-MS spectra of ***4,4'-Dicyanoazobenzene*** in the product mixture from the Hydrogenation of 4-Nitrobenzonitrile **(1o)**.

The Mass spectra of this compound has been previously reported.[11]

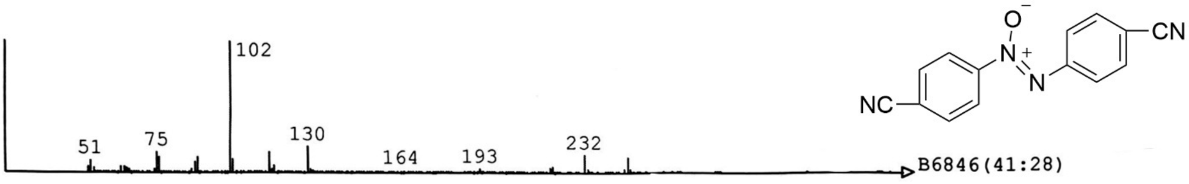

Figure S53. GC-MS spectra of ***4,4'-Dicyanoazoxybenzene*** in the product mixture from the Hydrogenation of 4-Nitrobenzonitrile **(1o)**.

The Mass spectra of this compound has been previously reported.[5]

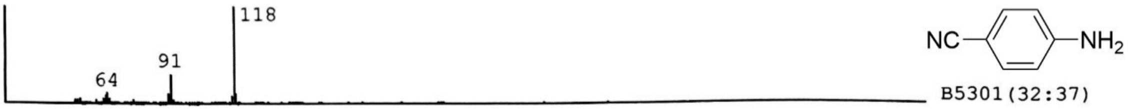

Figure S54. GC-MS spectra of ***4-Aminobenzonitrile*** in the product mixture from the Hydrogenation of 4-Nitrobenzonitrile **(1o)**.

The Mass spectra of this compound has been previously reported in NIST Mass Spectrometry Data Center. [2]

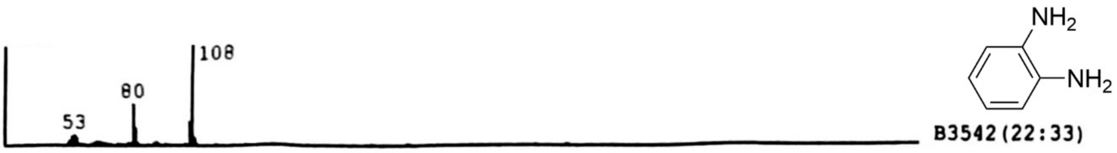

Figure S55. GC-MS spectra of ***1,2-Diaminobenzene*** in the product mixture from the Hydrogenation of 2-Nitroaniline **(1p)**.

The Mass spectra of this compound has been previously reported in NIST Mass Spectrometry Data Center. [2]

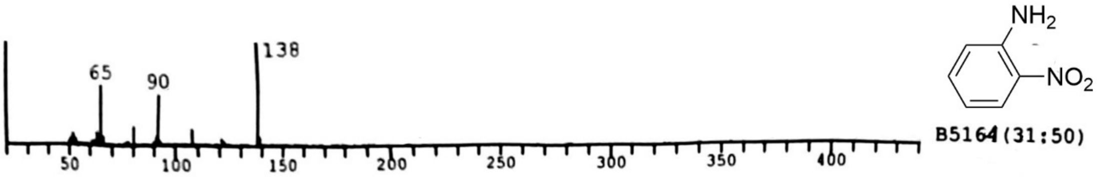

Figure S56. GC-MS spectra of ***2-Nitroaniline*** in the product mixture from the Hydrogenation of 2-Nitroaniline **(1p)**.

The Mass spectra of this compound has been previously reported in NIST Mass Spectrometry Data Center. [2]

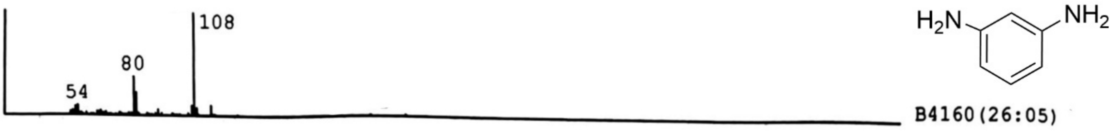

Figure S57. GC-MS spectra of ***1,3-Diaminobenzene*** in the product mixture from the Hydrogenation of 3-Nitroaniline **(1q)**.

The Mass spectra of this compound has been previously reported in NIST Mass Spectrometry Data Center. [2]

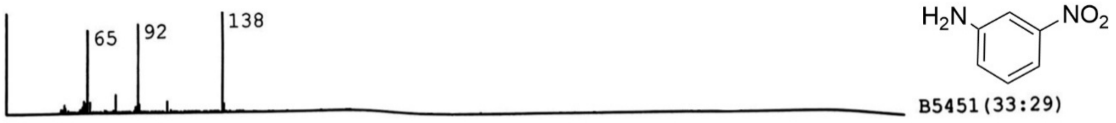

Figure S58. GC-MS spectra of ***3-Nitroaniline*** in the product mixture from the Hydrogenation of 3-Nitroaniline **(1q)**.

The Mass spectra of this compound has been previously reported in NIST Mass Spectrometry Data Center. [2]

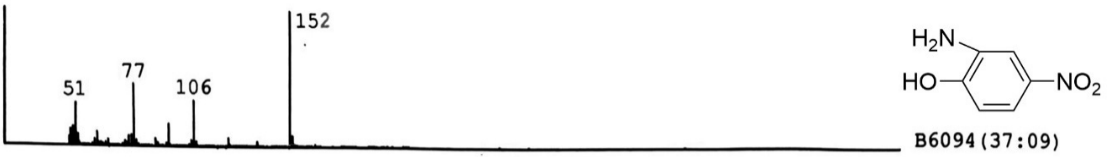

Figure S59. GC-MS spectra of ***2-Amino-4-nitrophenol*** in the product mixture from the Hydrogenation of 2-Amino-4-nitrophenol **(1r)**.

The Mass spectra of this compound has been previously reported on ChemicalBook (https://www.chemicalbook.com/SpectrumEN_99-57-0_MS.htm).

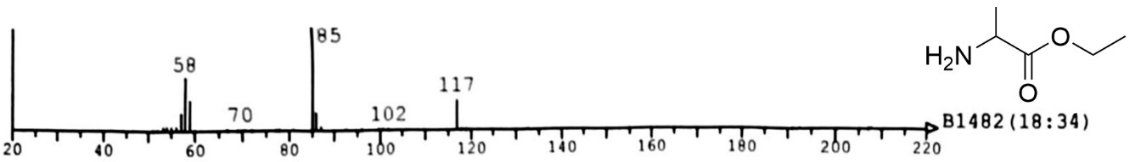

Figure S60. GC-MS spectra of ***Ethyl alaninate*** in the product mixture from the Hydrogenation of ethyl 2-nitropropionate **(1s)**.

The Mass spectra of this compound has been previously reported.[5]

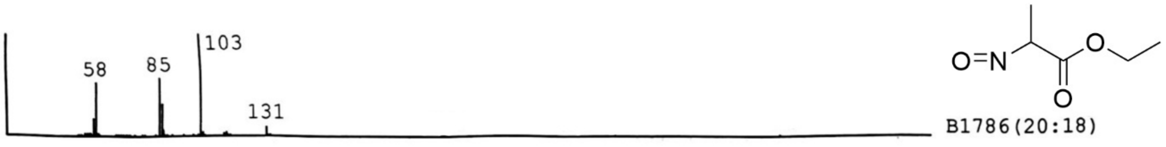

Figure S61. GC-MS spectra of ***Ethyl 2-nitrosopropanoate*** in the product mixture from the Hydrogenation of 2-nitropropionate **(1s)**.

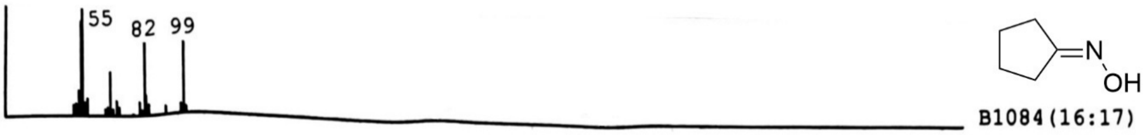

Figure S62. GC-MS spectra of ***Cyclopentanone oxime*** in the product mixture from the Hydrogenation of Nitrocyclopentane **(1t)**.

The Mass spectra of this compound has been previously reported in NIST Mass Spectrometry Data Center. [2]

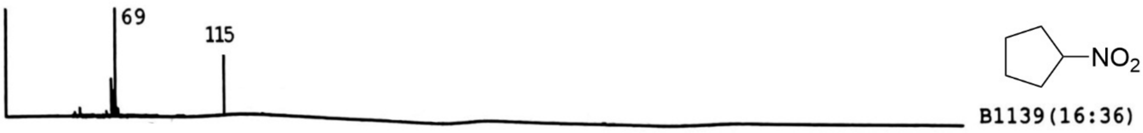

Figure S63. GC-MS spectra of ***Nitrocyclopentane*** in the product mixture from the Hydrogenation of Nitrocyclopentane **(1t)**.

The Mass spectra of this compound has been previously reported in NIST Mass Spectrometry Data Center. [2]

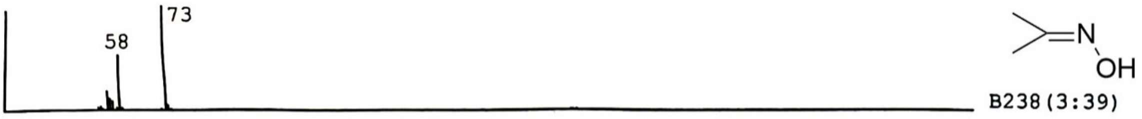

Figure S64. GC-MS spectra of ***Propan-2-one oxime*** in the product mixture from the Hydrogenation of 2-Nitropropane **(1u)**.

The Mass spectra of this compound has been previously reported in NIST Mass Spectrometry Data Center. [2]

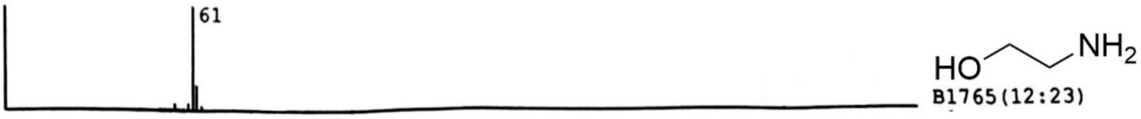

Figure S65. GC-MS spectra of ***2-Aminoethanol*** in the product mixture from the Hydrogenation of 2-Nitroethanol **(1v)**.

The Mass spectra of this compound has been previously reported in NIST Mass Spectrometry Data Center. [2]

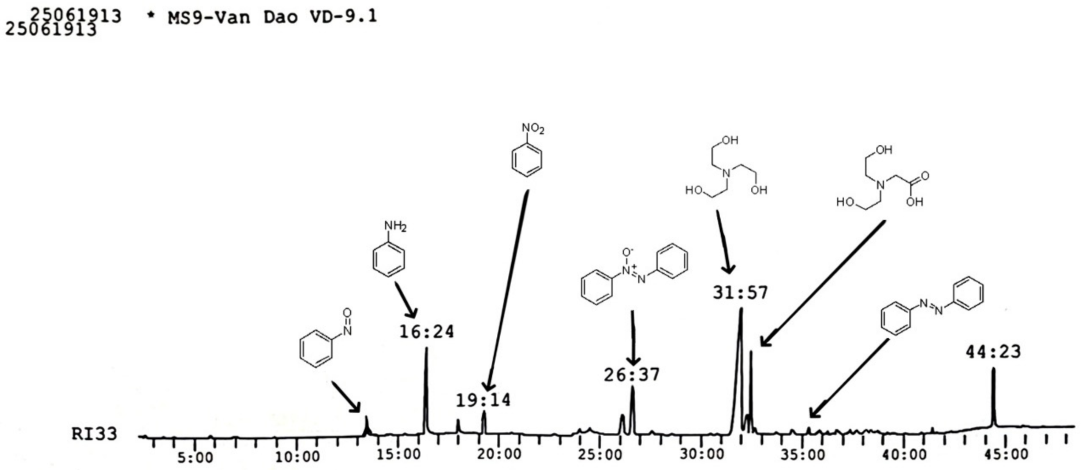

Figure S66. GC-MS spectra of nitrobenzene reduction reaction after 9 hours of irradiation

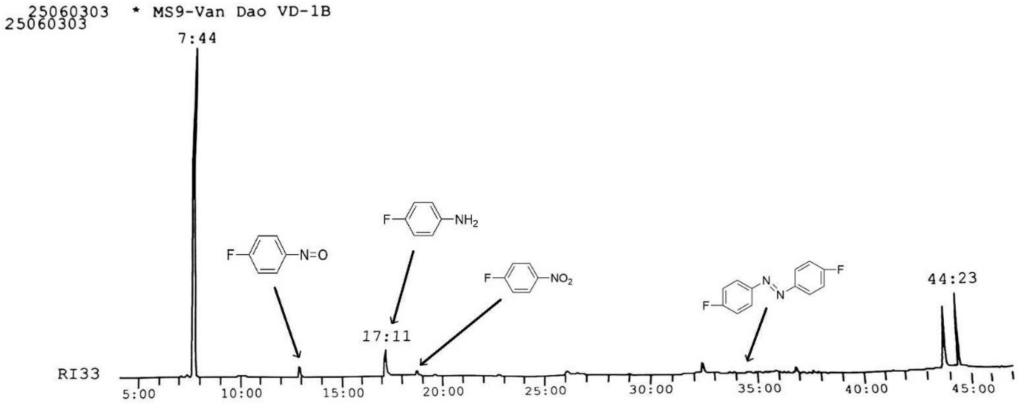

Figure S67. GC-MS spectra of the product mixture of the Hydrogenation of 1-Fluoro-4-nitrobenzene **(1a)**

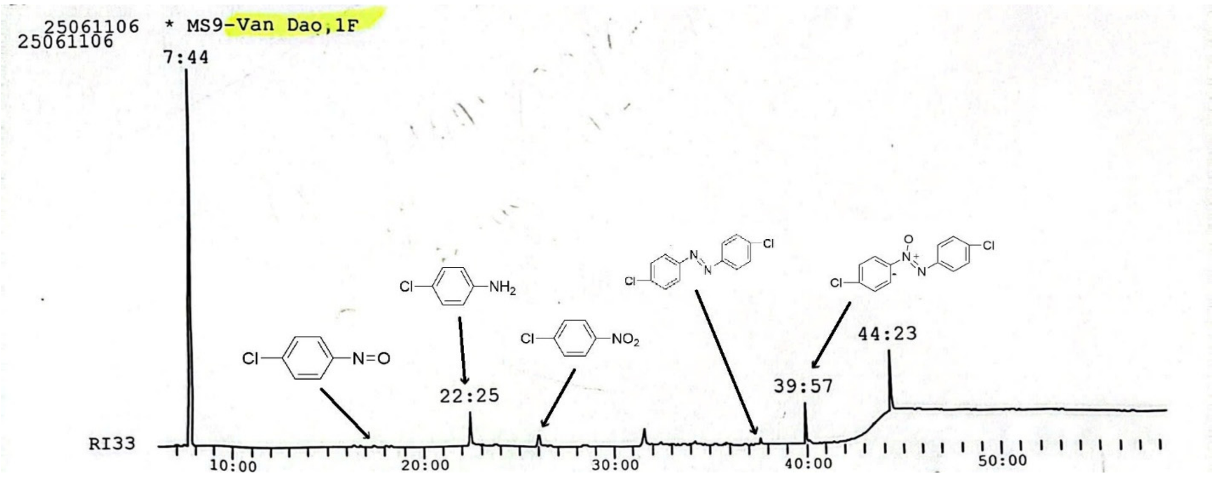

Figure S68. GC-MS spectra of the product mixture of the Hydrogenation of 1-Chloro-4-nitrobenzene **(1b)**

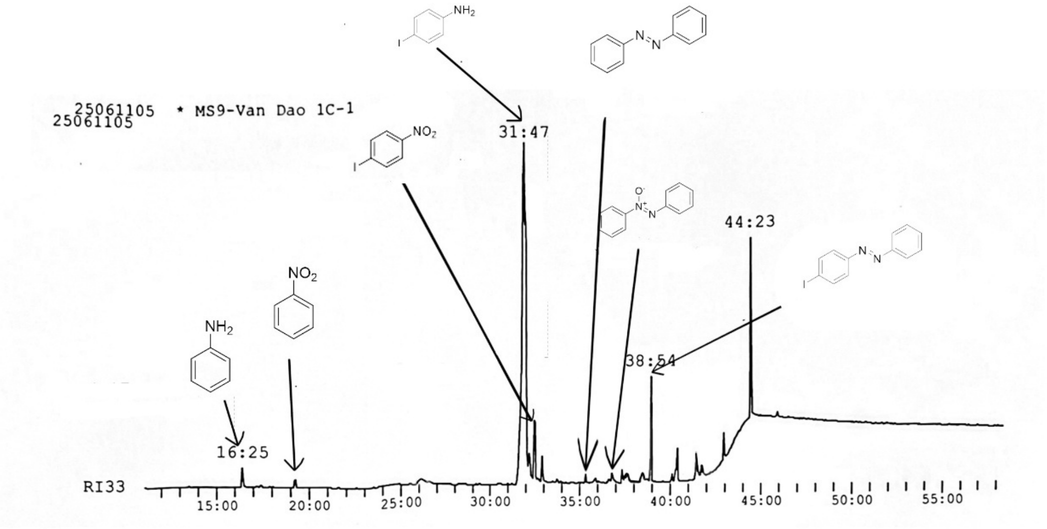

Figure S69. GC-MS spectra of the product mixture of the Hydrogenation of 1-Iodo-4-nitrobenzene **(1c)**

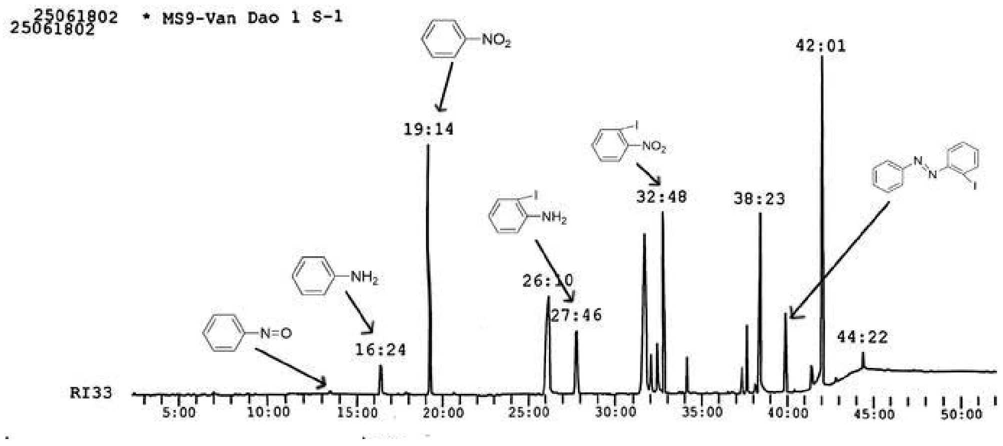

Figure S70. GC-MS spectra of the product mixture of the Hydrogenation of 1-Iodo-2-nitrobenzene **(1d)**

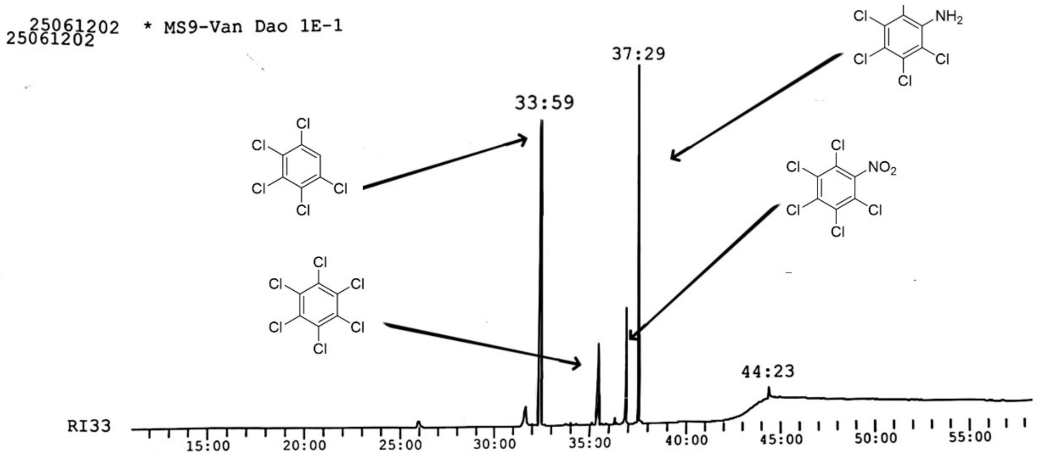

Figure S71. GC-MS spectra of the product mixture of the Hydrogenation of Quinozene/Pentachloronitrobenzene **(1e)**

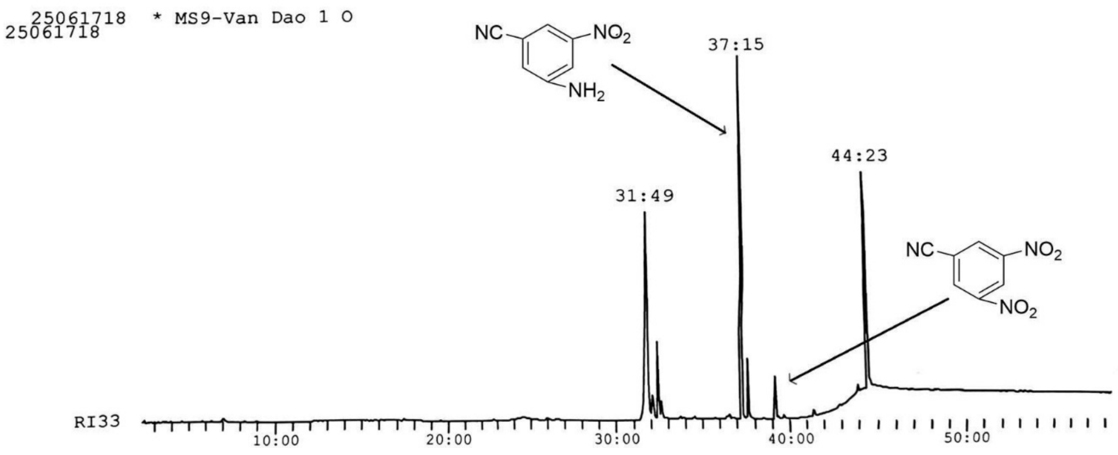

Figure S72. GC-MS spectra of the product mixture of the Hydrogenation of 3,5-Dinitrobenzonitrile **(1f)**

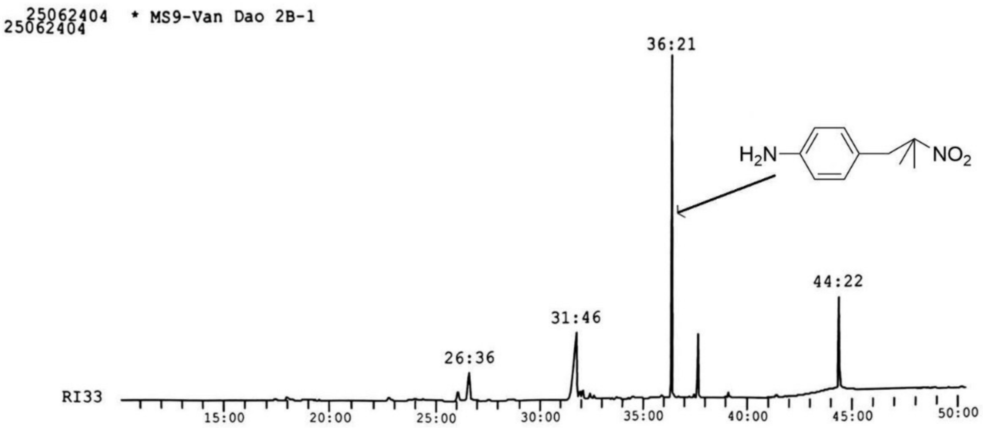

Figure S73. GC-MS spectra of the product mixture of the Hydrogenation of 2-Nitro-2-(4-nitrobenzyl)propane **(1g)**

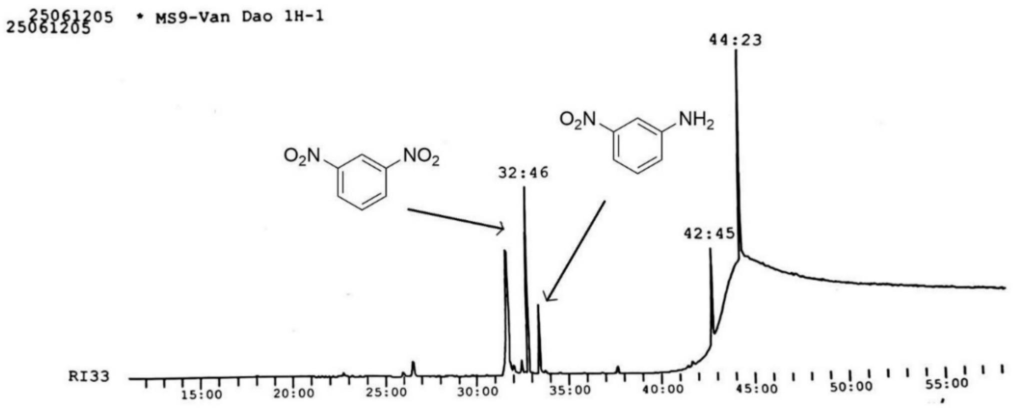

Figure S74. GC-MS spectra of the product mixture of the Hydrogenation of 1,3-Dinitrobenzene **(1h)**

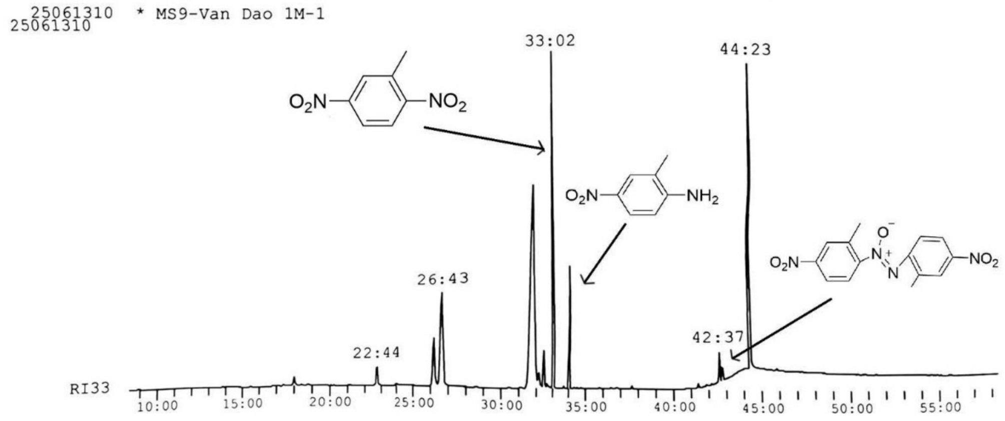

Figure S75. GC-MS spectra of the product mixture of the Hydrogenation of 2,5-Dinitrotoluen **(1i)**

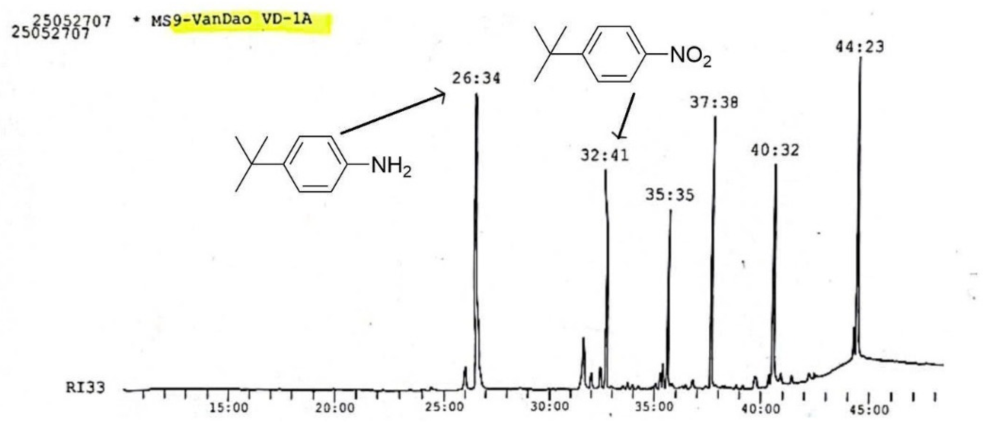

Figure S76. GC-MS spectra of the product mixture of the Hydrogenation of 1-tert-Butyl-4-nitrobenzene **(1j)**

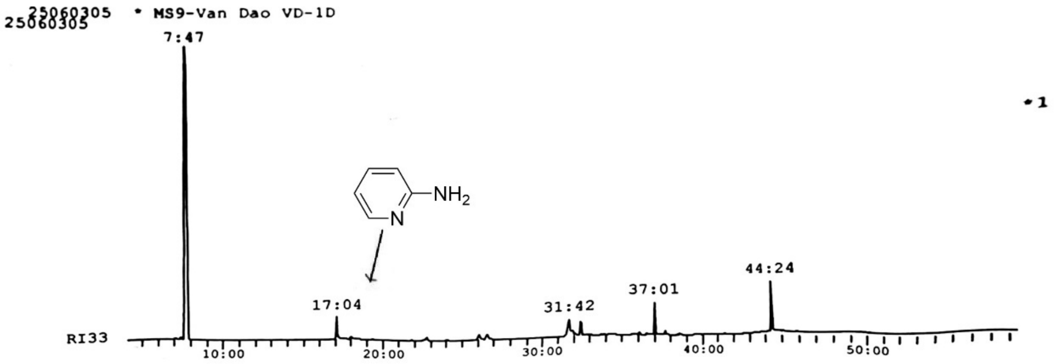

Figure S77. GC-MS spectra of the product mixture of the Hydrogenation of 2-Nitropyridine **(1k)**

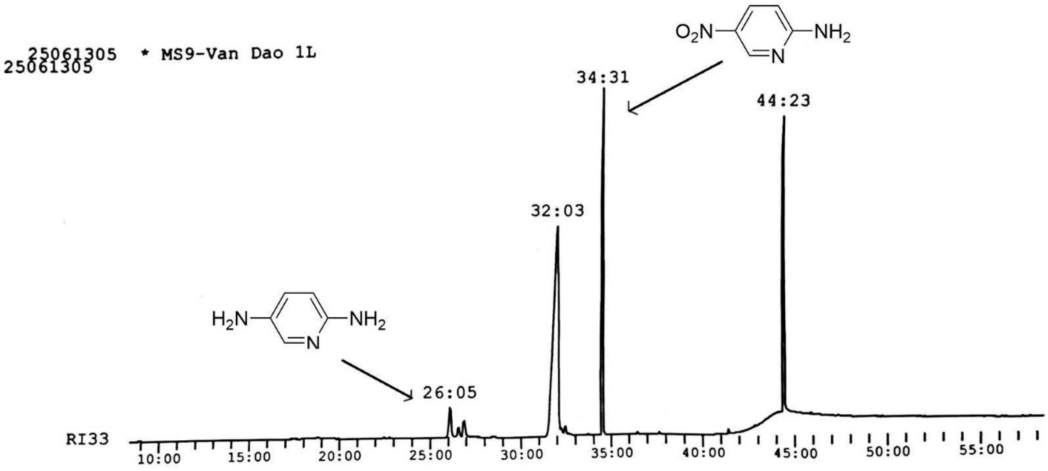

Figure S78. GC-MS spectra of the product mixture of the Hydrogenation of 2-Amino-5-Nitropyridine **(1l)**

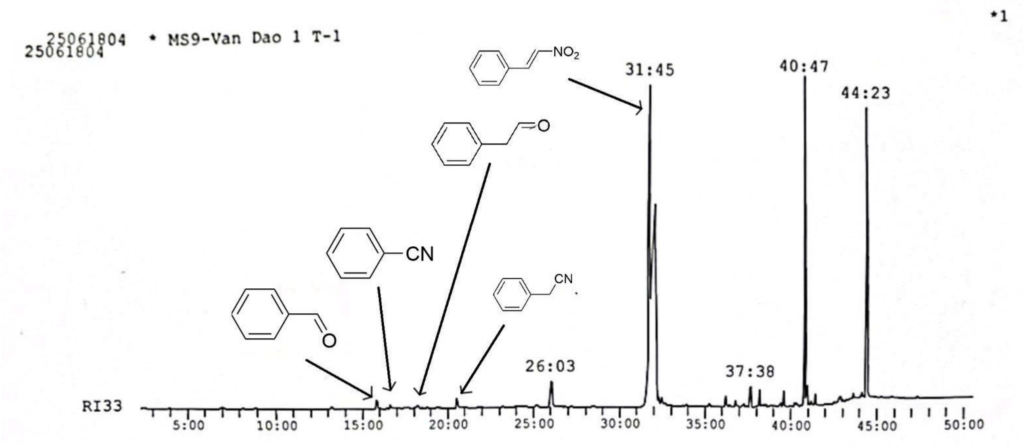

Figure S79. GC-MS spectra of the product mixture of the Hydrogenation of Trans-β-nitrostyrene **(1m)**

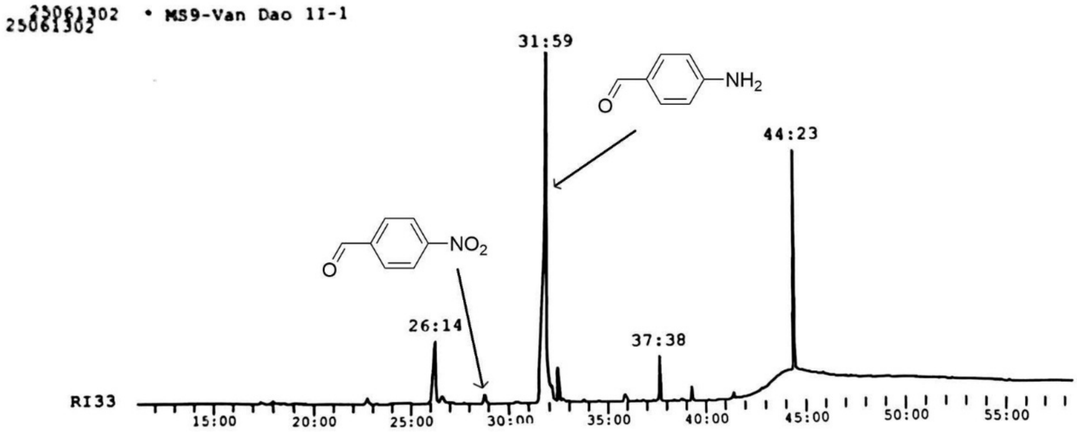

Figure S80. GC-MS spectra of the product mixture of the Hydrogenation of 4-Nitrobenzaldehyde **(1n)**

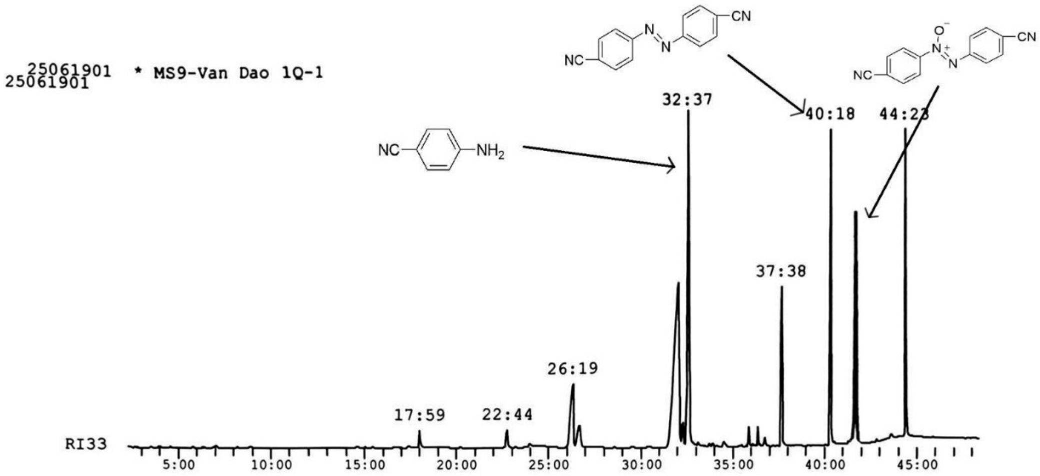

Figure S81. GC-MS spectra of the product mixture of the Hydrogenation of 4-Nitrobenzonitrile **(1o)**

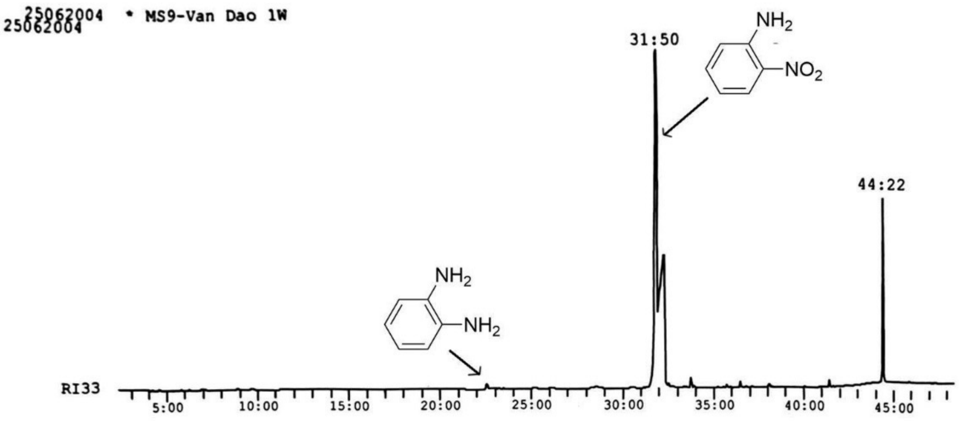

Figure S82. GC-MS spectra of the product mixture of the Hydrogenation of 2-Nitroaniline **(1p)**

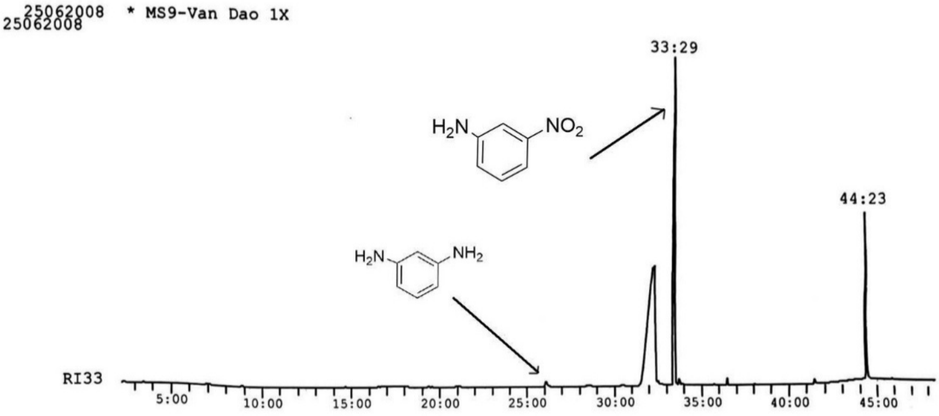

Figure S83. GC-MS spectra of the product mixture of the Hydrogenation of 3-Nitroaniline **(1q)**

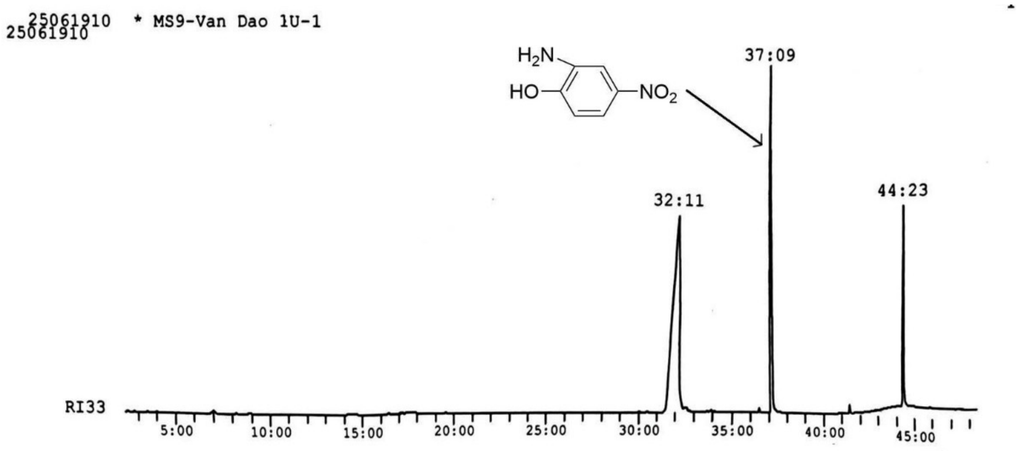

Figure S84. GC-MS spectra of the product mixture of the Hydrogenation of 2-Amino-4-nitrophenol **(1r)**

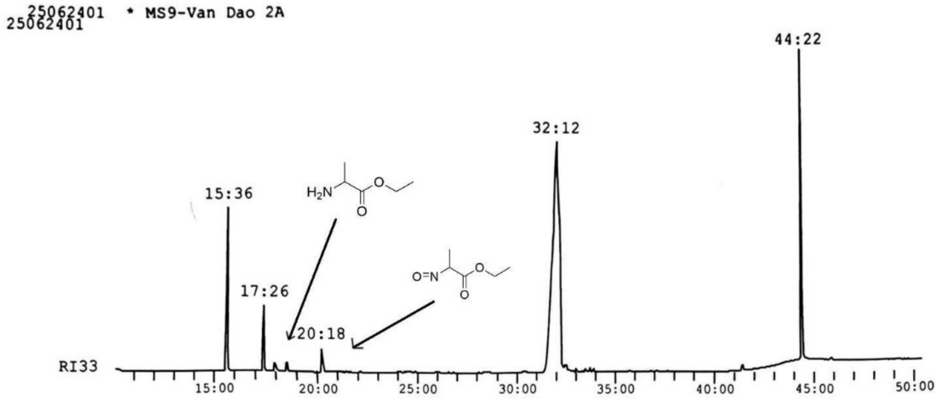

Figure S85. GC-MS spectra of the product mixture of the Hydrogenation of ethyl 2-nitropropionate **(1t)**

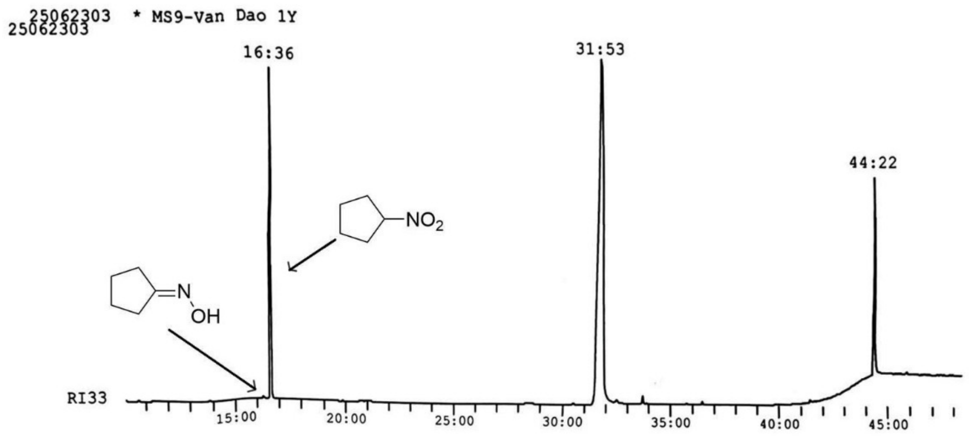

Figure S86. GC-MS spectra of the product mixture of the Hydrogenation of Nitrocyclopentane **(1u)**

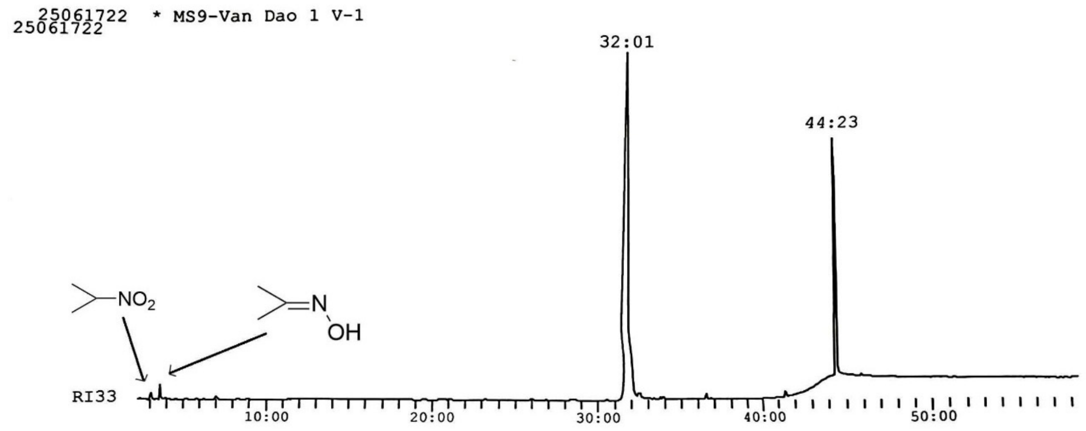

Figure S87. GC-MS spectra of the product mixture of the Hydrogenation of 2-Nitropropane **(1v)**

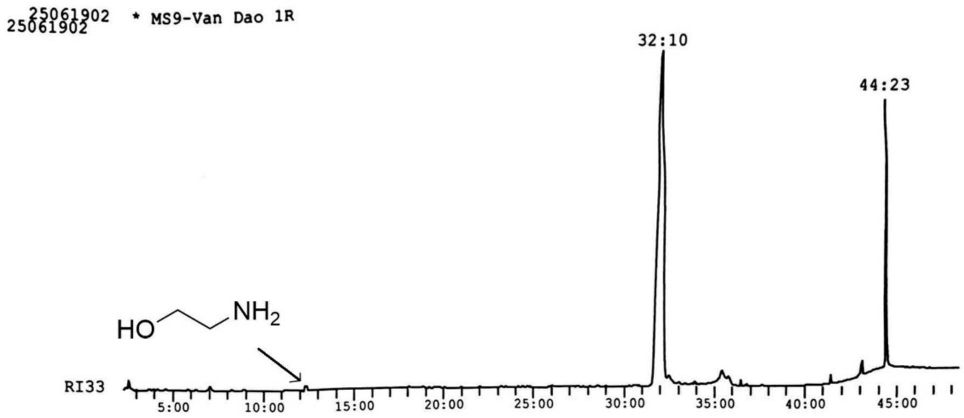

Figure S88. GC-MS spectra of the product mixture of the Hydrogenation of 2-Nitroethanol **(1w)**

5. UV-Vis Spectroscopy

UV–vis spectra were obtained by an AvaSpec-2048 UV–vis spectrometer (Avantes) with a fiber-optical probe at 300 K and ambient pressure. Photocatalysts dissolved in acetonitrile was added in different concentration until strong and good detectable absorption bonds appeared. This approach was applied on different catalysts (see figure below). For the time-resolved UV-Vis study, the immersion probe was dipped into a 10 mL Schlenk tube containing the components of a typical experiment under the irradiation of LED 390nm in Argon condition. The reaction mixture suitable concentration was determined by the dilution of the typical reaction mixture until strong and good detectable absorption bonds appeared. The reaction was turned off and the UV-Vis spectroscopy was recorded in every desired timestamps. The spectra were recorded in a range of 300 – 1000 nm.

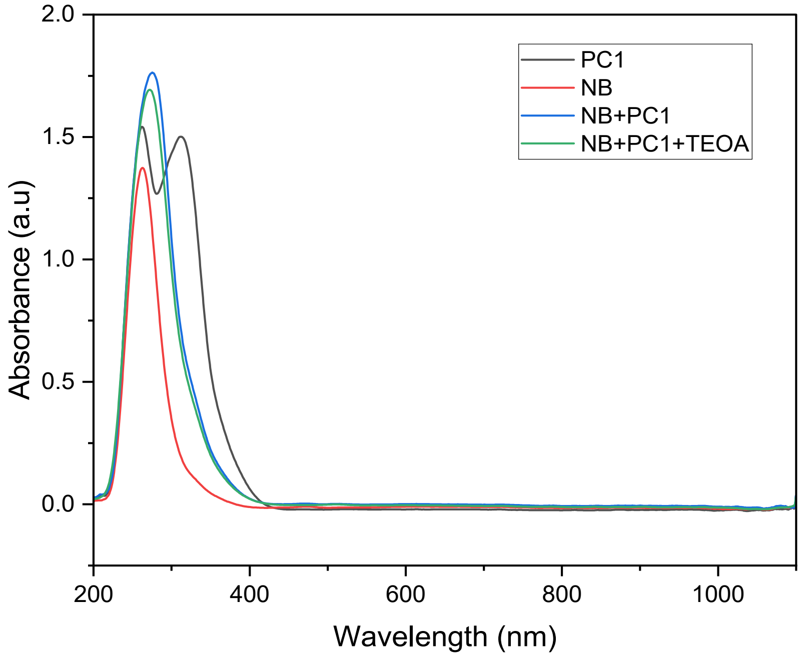

Figure S89. UV–Vis absorption spectra of individual components and photocatalytic mixtures recorded in acetonitrile at 300 K and ambient pressure: (black) PC1 (4.16×10^{-6} M); (red) nitrobenzene (NB, 8.3×10^{-5} M); (blue) NB (4.2×10^{-5} M) + PC1 (2.8×10^{-3} M); (green) NB (2.1×10^{-5} M) + PC1 (1.04×10^{-5} M) + TEOA (6.25×10^{-5} M).

6. Fluorescence Spectroscopy

Fluorescence Spectroscopy (FL) emission spectra were measured at room temperature on a fluorescence spectrophotometer (Cary Eclipse, Varian, USA) using 370, 390, 440 and 467 nm as the excitation light source's wavelength.

To determine the quenching capabilities of the excited state PC by TEOA and NB, a series of fluorescence measurements was performed using different components of the reaction system. Stock solutions of NB (0.167 M), TEOA (0.5 M), and PC1 (0.025 M) were prepared and distributed into 4 mL cuvettes in volumes according to the experimental design.

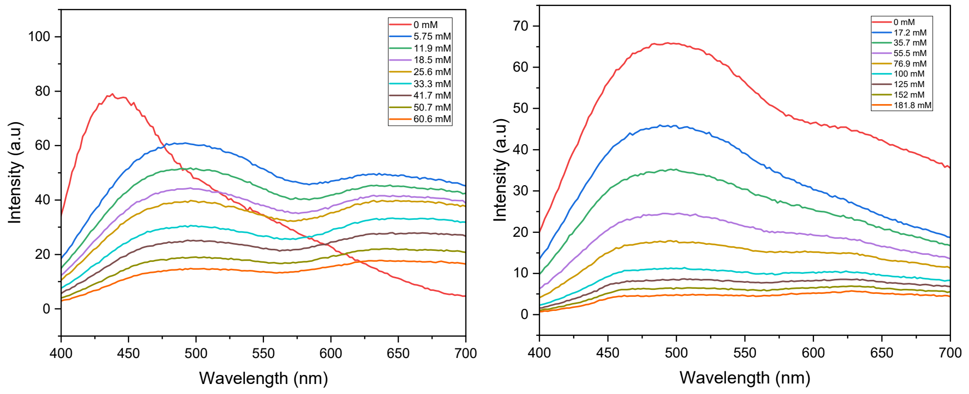

Figure S90. Fluorescence emission spectrum of the PC1 after the addition of NB (a) and TEOA (b) measured in acetonitrile at standard conditions with the excited wavelength of 370nm.

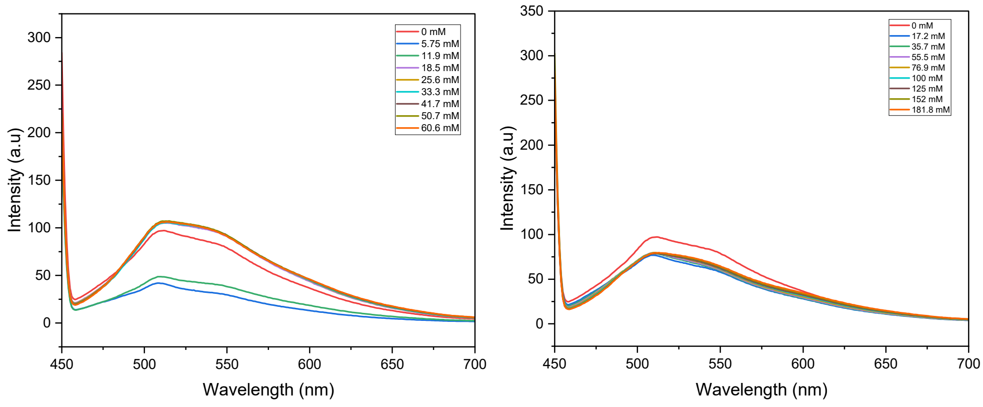

Figure S91. Fluorescence emission spectrum of the PC1 after the addition of NB (a) and TEOA (b) measured in acetonitrile at standard conditions with the excited wavelength of 440nm.

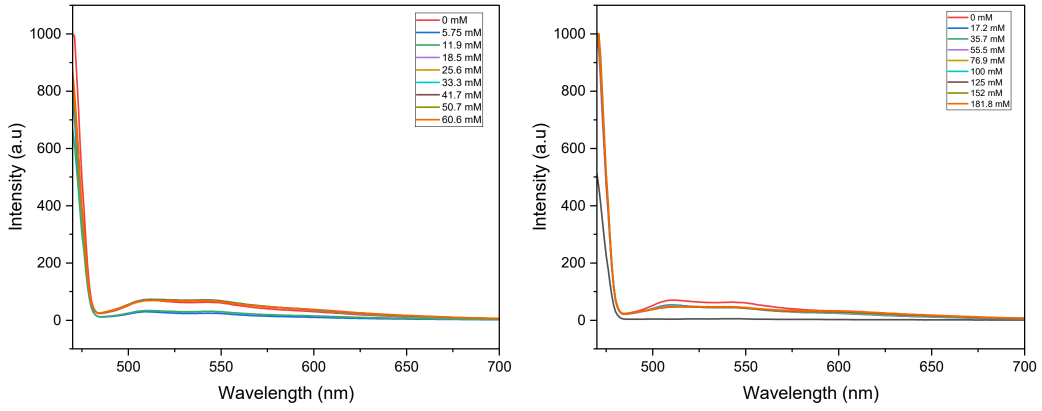

Figure S92. Fluorescence emission spectrum of the PC1 after the addition of NB (a) and TEOA (b) measured in acetonitrile at standard conditions with the excited wavelength of 467nm.

7. EPR Spectroscopy

EPR spectra were recorded on an X-band Bruker EMX CW-micro EPR spectrometer equipped with an ER4119HS high-sensitivity resonator using a microwave frequency of $\nu\approx$ 9.7 GHz, a microwave power of 6.3 mW, a modulation frequency of 100 kHz and a modulation amplitude of 1 G, a scanning number of 1 and sweeping time of 30 s. The $h\nu = g\beta B_0$ equation was used to calculate g values with ν and B_0 being the microwave frequency and resonance field, respectively. 2,2-Diphenyl-1-picrylhydrazyl (DPPH) was used as a standard ($g = 2.0036 \pm 0.0004$) for calibration of the g value. The EPR spectra were simulated with MatlabR2023a using the EasySpin-5.2.36 module.

EPR spin trapping experiments with and without 5,5-dimethyl-1-pyrroline N-oxide (DMPO) were performed as following:

Determination of the formation of radical during the reaction

Using a 10 mL vial, photocatalyst PC1 (8.4 mg, 0.025 mmol, 5 mol%), starting Nitrobenzene (51.3 µl, 0.5 mmol, 1.0 eq), and TEOA (233.77 mg, 1.5 mmol, 4.0 eq) were added in 2 mL CH_3CN (dried over molecular sieves before use. Approximately 1 mL of the reaction solution was transferred to an EPR flat cell, and EPR spectra were recorded in dark and under irradiation (40 W Kessil lamp 390nm) for different time periods at room temperature.

Spin trapping with 5,5-dimethylpyrroline N-oxide (DMPO)

Using a 10 mL vial, photocatalyst PC1 (8.4 mg, 0.025 mmol, 5 mol%), starting substrate Nitrobenzene (51.3 µl, 0.5 mmol, 1.0 eq), and TEOA (233.77 mg, 1.5 mmol, 4.0 eq) were added in 2 mL CH_3CN (dried over molecular sieves before use). Approximately 1 mL of the reaction solution was taken out and mixed with 10 µL DMPO. Then about 50 µL of this mixture was transferred into a glass microcapillary tube (Hirschmann), and EPR spectra were recorded in dark and under irradiation (40 W Kessil lamp 390nm) for 1 hour at room temperature.

Table S5. EPR simulation parameters of different radicals

Radicals	g_{iso}	Hyperfine coupling constants (G)		
		N	H^{β}	H^{γ}
DMPO-$O_2^{\bullet-}$ or DMPO-OOH	2.0068	12.80	10.73	–
DMPO-C centered	2.0068	15.1	21.0	_
DMPO-OPh	2.0068	12.85	10.66	1.32

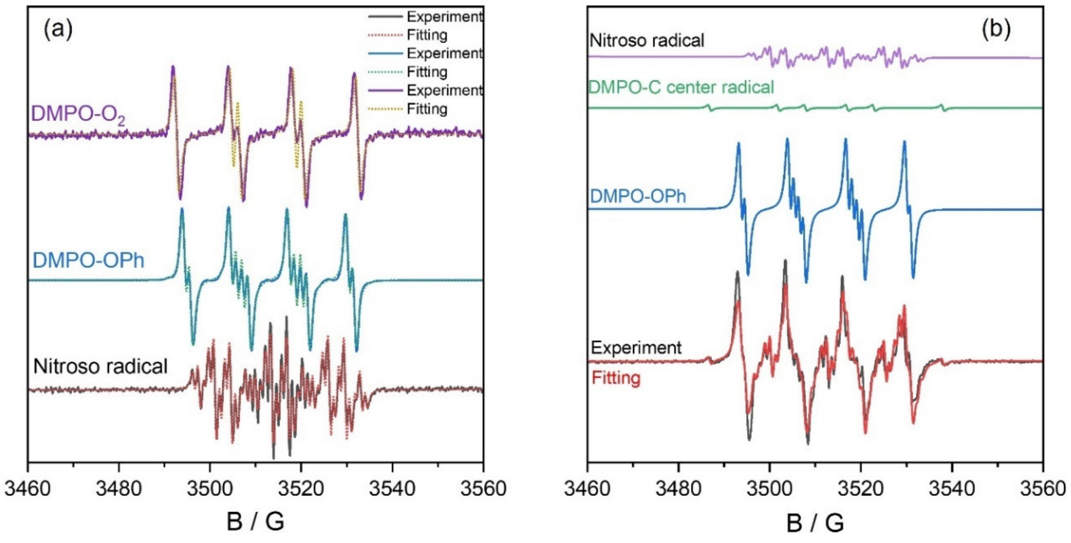

Figure S93. EPR spectra of (a) nitrosobenzene radical and DMPO-OPh (O-centered organic radical derived from nitrobenzene) spin adduct formed upon irradiation of solution S1 (standard reaction mixture without PC1) and solution S2 (standard reaction mixture without TEOA) with DMPO, respectively; and DMPO-O_2 spin adduct formed after adding DMPO into the reaction mixture under dark condition, together with their simulated spectra; (b) EPR spectra DMPO-containing S1 solution recorded upon irradiation, together with fitting spectra and all simulated spectral components (40 W Kessil lamp 390nm).

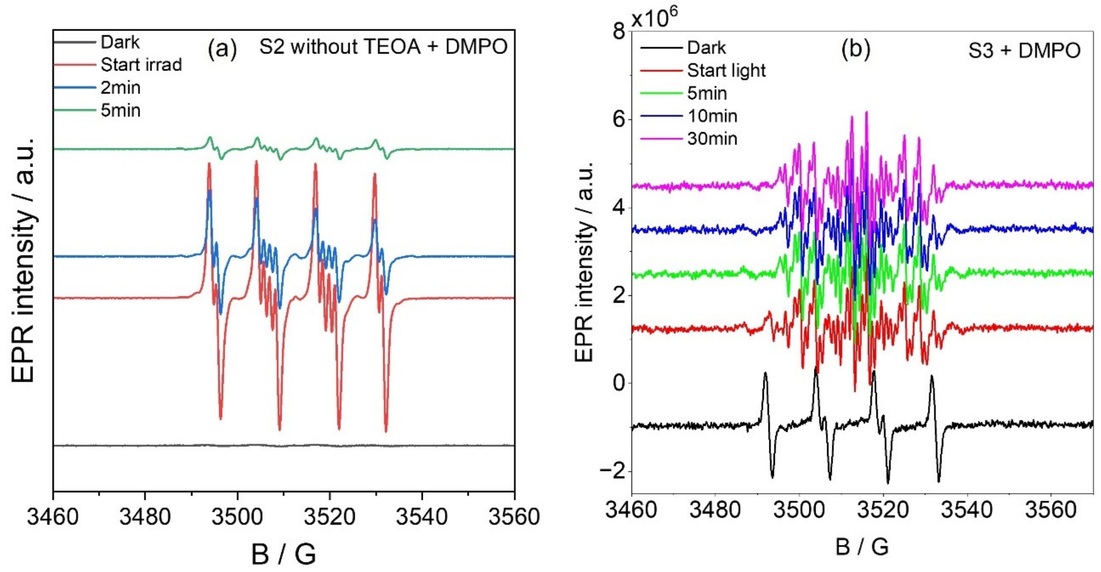

Figure S94. EPR spectra of (a) solution S2 (standard reaction mixture without TEOA) and (b) solution S3 (standard reaction mixture) in the presence of DMPO before and during irradiation (40 W Kessil lamp 390nm).

8. NMR Spectra of the Photocatalysts

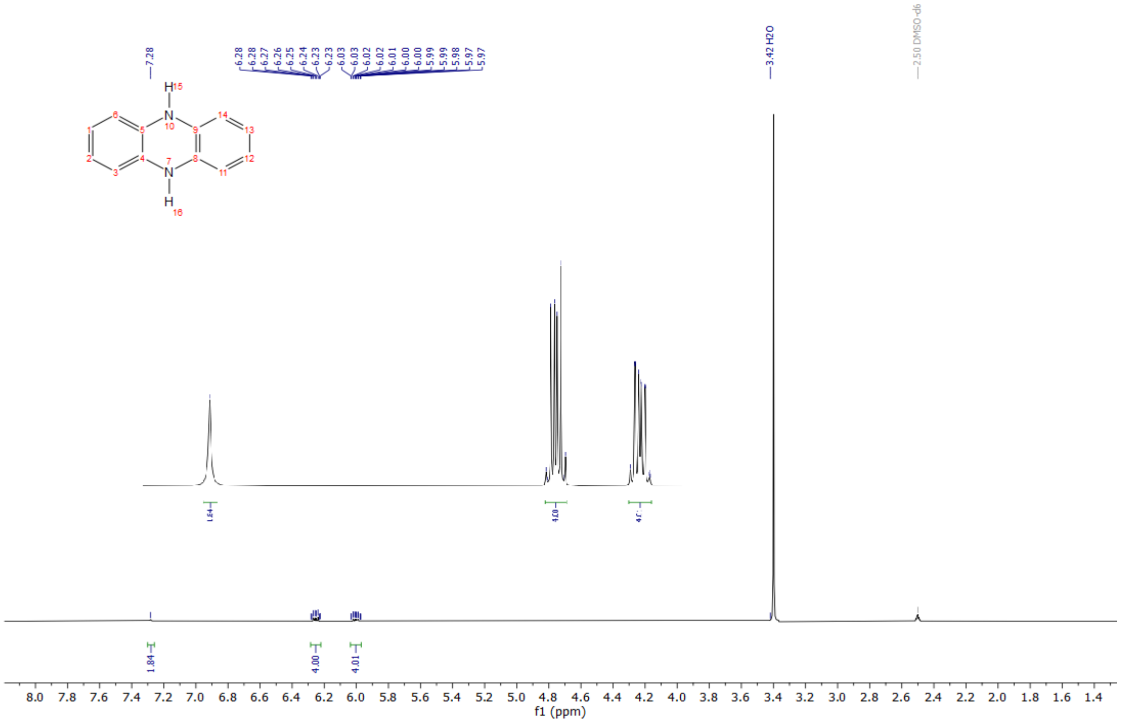

Figure S95. ^{1}H NMR (300 MHz, DMSO-d6) spectrum of N,N'-dihydrophenazine.

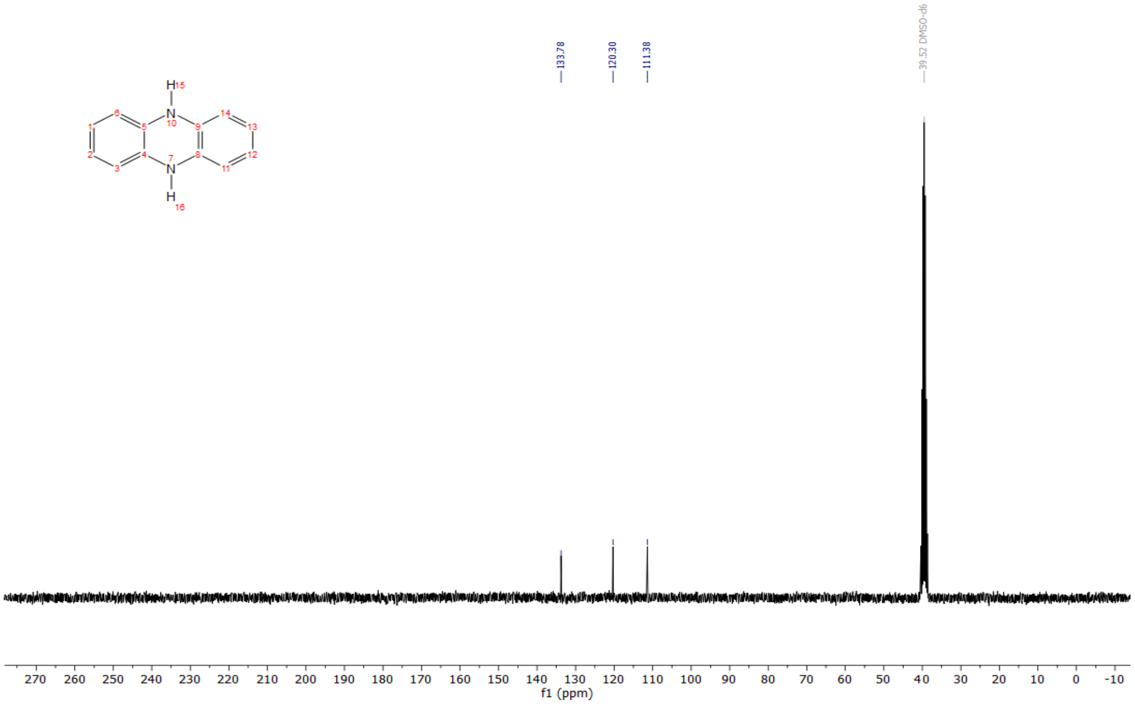

Figure S96. ^{13}C NMR (75 MHz, DMSO-d6) spectrum of N,N'-dihydrophenazine.

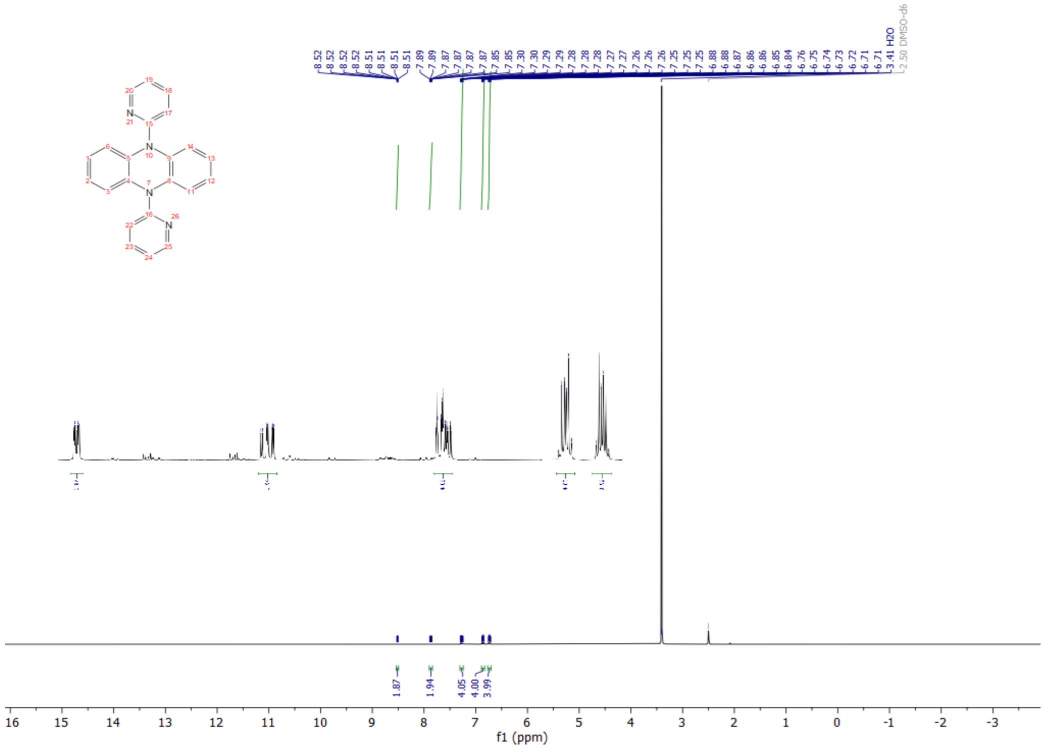

Figure S97. ^{1}H NMR (400 MHz, DMSO-d6) spectrum of PC1.

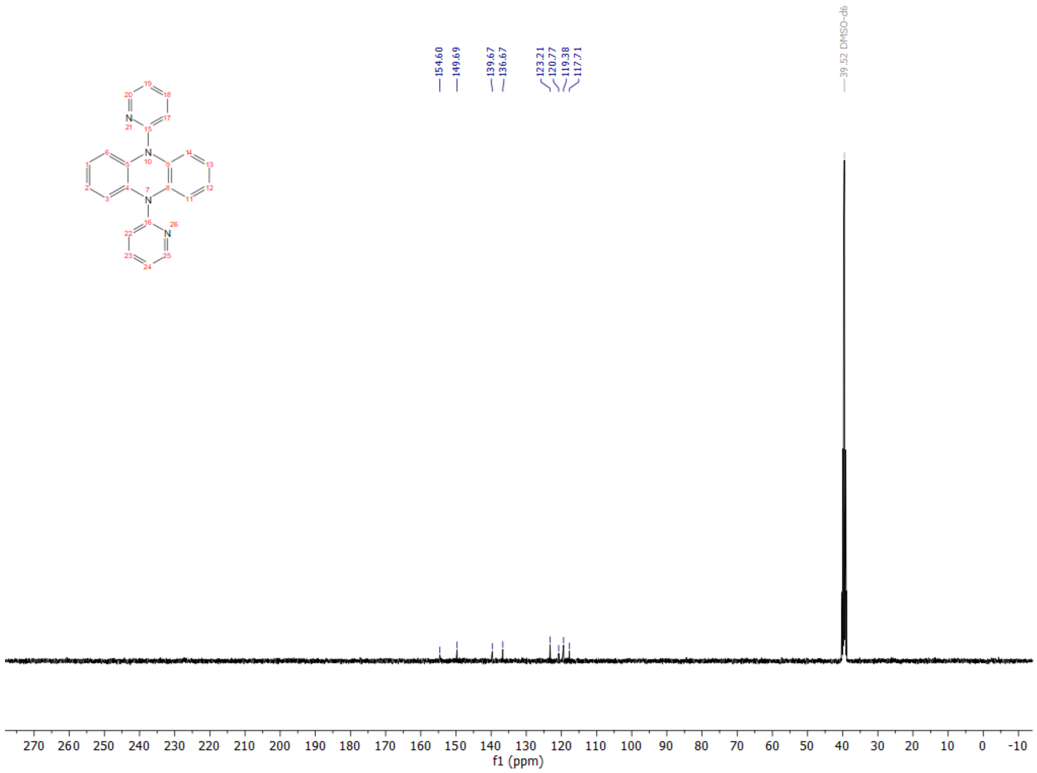

Figure S98. ^{13}C NMR (75 MHz, DMSO-d6) spectrum of PC1.

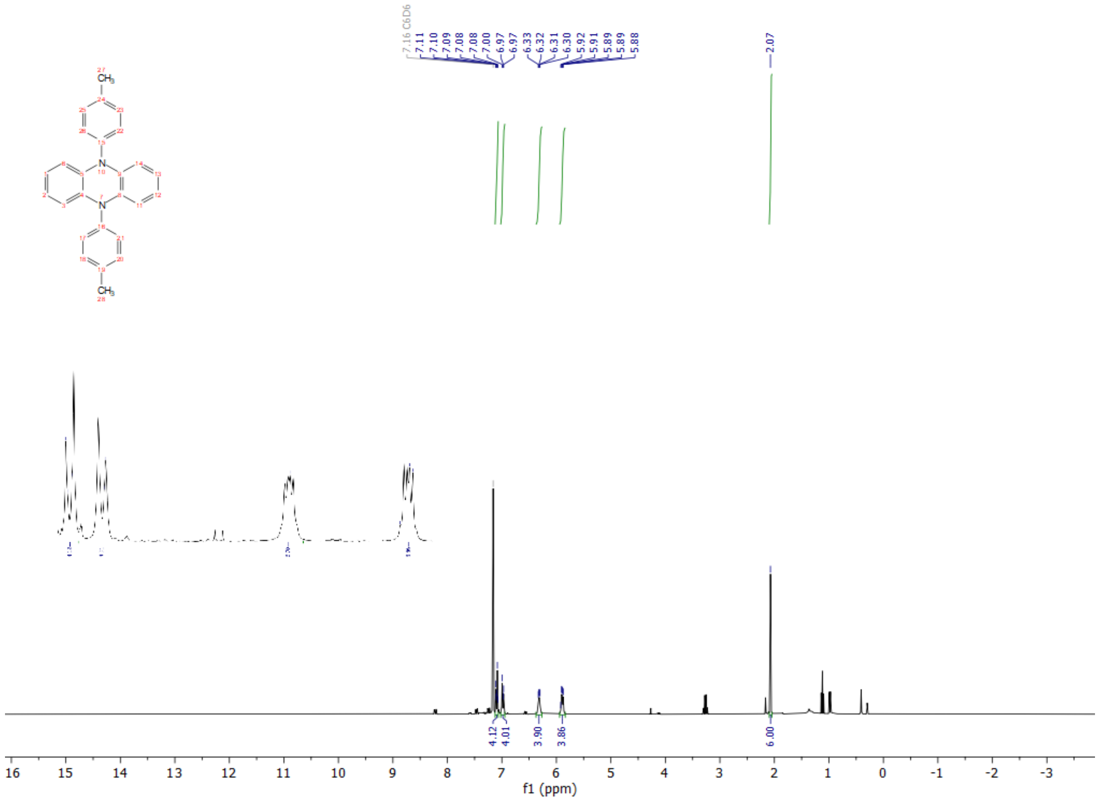

Figure S99. ^{1}H NMR (300 MHz, C_6H_6-d6) spectrum of PC2.

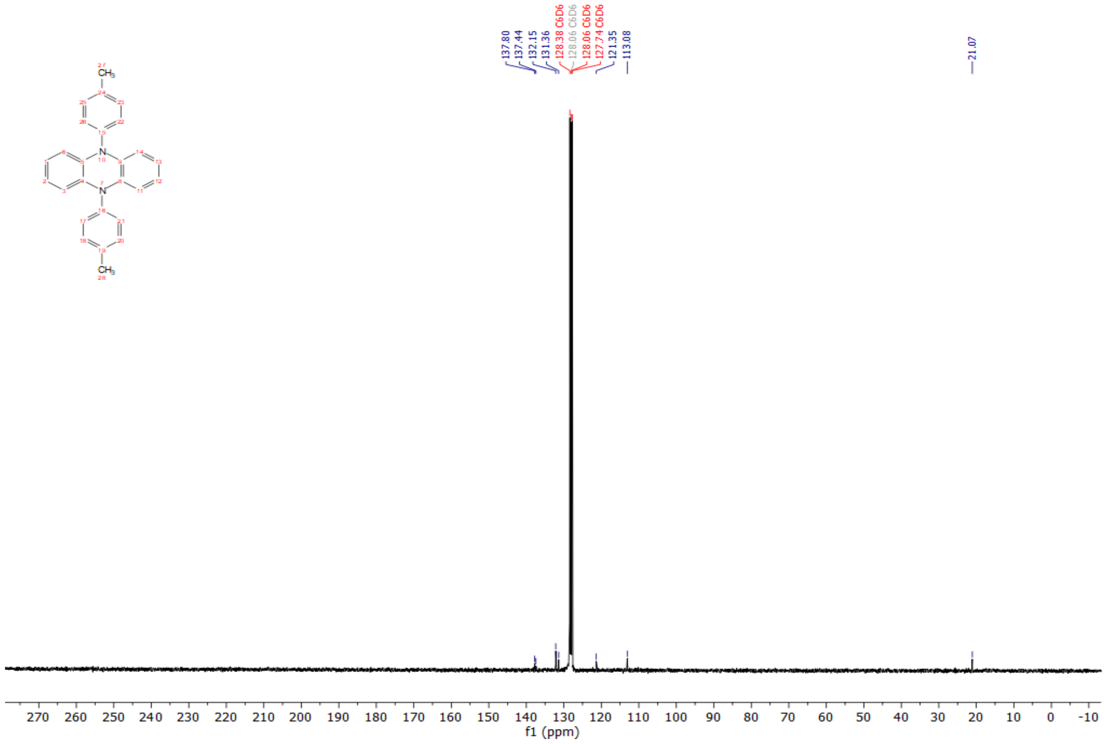

Figure S100. ^{13}C NMR (75 MHz, C_6H_6-d6) spectrum of PC2.

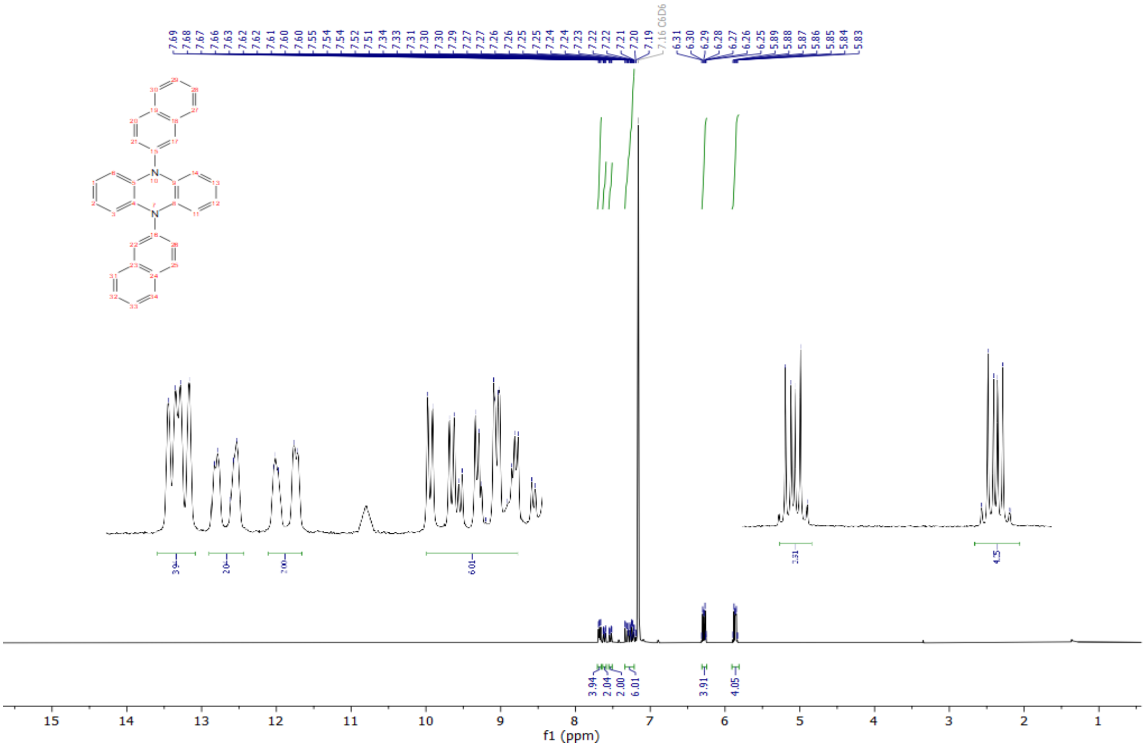

Figure S101. ^{1}H NMR (300 MHz, C_6H_6-d6) spectrum of PC3.

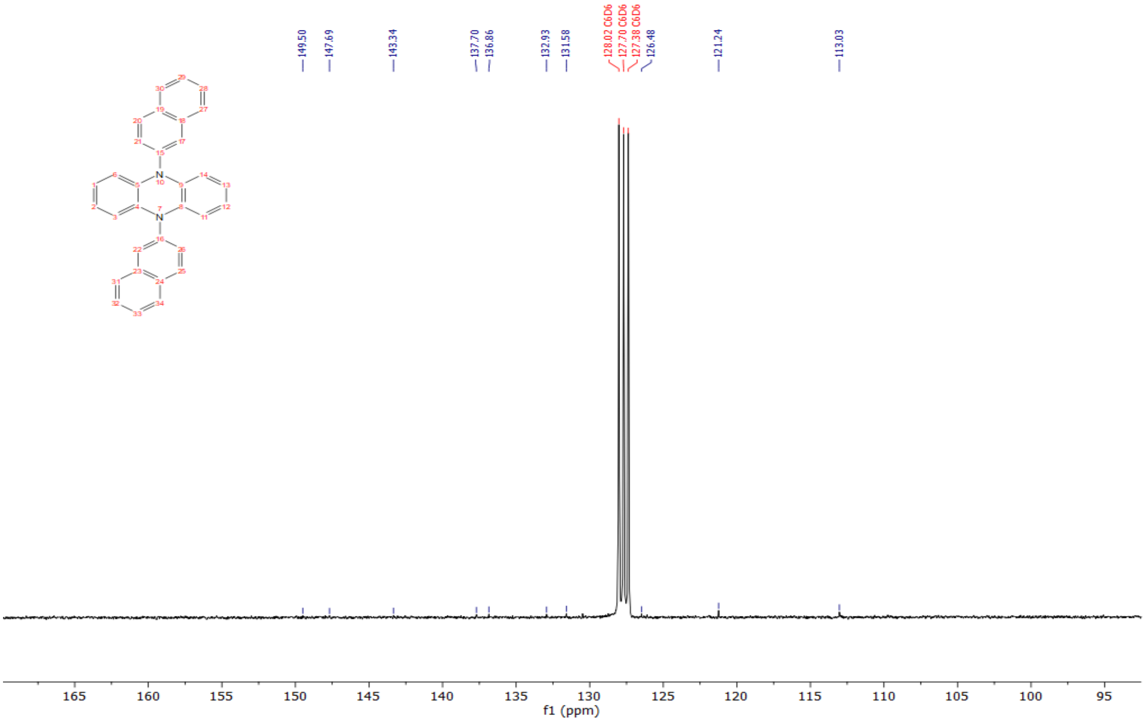

Figure S102. ^{13}C NMR (75 MHz, C_6H_6-d6) spectrum of PC3.

References

[1] P. Phan Huyen Quyen, N. Hagmeyer, T. H. Vuong, A. Prudlik, R. Francke, B. Dietzek-Ivanšić, E. Mejía, 'Janus-type photo-redox properties and catalytic applications of 5,10-dihydrophenazine derivatives', *Organic Chemistry Frontiers* **2025**, *12*, 6798–6819.

[2] W. E. Wallace, A. S. Moorthy, 'NIST Mass Spectrometry Data Center standard reference libraries and software tools: Application to seized drug analysis', *Journal of Forensic Sciences* **2023**, *68*, 1484–1493.

[3] Z. Yuan, L. Huang, Y. Liu, Y. Sun, G. Wang, X. Li, J. A. Lercher, Z. Zhang, 'Synergy of Oxygen Vacancies and Base Sites for Transfer Hydrogenation of Nitroarenes on Ceria Nanorods', *Angewandte Chemie International Edition* **2024**, *63*, e202317339.

[4] Y. Long, J. Qin, J. Ma, Lanzhou University, China, **2021**.

[5] J. Wang, X. Yu, C. Shi, D. Lin, J. Li, H. Jin, X. Chen, S. Wang, 'Iron and Nitrogen Co-Doped Mesoporous Carbon-Based Heterogeneous Catalysts for Selective Reduction of Nitroarenes', *Advanced Synthesis & Catalysis* **2019**, *361*, 3525–3531.

[6] T. A. Lutz, P. Spanner, K. T. Wanner, 'A general approach to substituted diphenyldiazenes', *Tetrahedron* **2016**, *72*, 1579–1589.

[7] J. H. Advani, K. Ravi, D. R. Naikwadi, H. C. Bajaj, M. B. Gawande, A. V. Biradar, 'Bio-waste chitosan-derived N-doped CNT-supported Ni nanoparticles for selective hydrogenation of nitroarenes', *Dalton Transactions* **2020**, *49*, 10431–10440.

[8] H. Kakuta, R. Fukai, Z. Xiaoxia, F. Ohsawa, T. Bamba, K. Hirata, A. Tai, 'Identification of urine metabolites of TFAP, a cyclooxygenase-1 inhibitor', *Bioorganic & Medicinal Chemistry Letters* **2010**, *20*, 1840–1843.

[9] E. M. Di Tommaso, M. Walther, A. Staubitz, B. Olofsson, 'ortho-Functionalization of azobenzenes via hypervalent iodine reagents', *Chemical Communications* **2023**, *59*, 5047–5050.

[10] V. V. Patil, G. S. Shankarling, 'Steric-Hindrance-Induced Regio- and Chemoselective Oxidation of Aromatic Amines', *The Journal of Organic Chemistry* **2015**, *80*, 7876–7883.

[11] Y. Takeda, S. Okumura, S. Minakata, 'A Practical Synthesis of Azobenzenes through Oxidative Dimerization of Aromatic Amines Using tert-Butyl Hypoiodite', *Synthesis* **2013**, *45*, 1029–1033.
